# The Impact of Human Mobility on HIV Transmission in Kenya

**DOI:** 10.1371/journal.pone.0142805

**Published:** 2015-11-24

**Authors:** Augustino Isdory, Eunice W. Mureithi, David J. T. Sumpter

**Affiliations:** 1 Department of Mathematics, University of Dar es Salaam, Dar es Salaam, Tanzania; 2 Department of Mathematics, Uppsala University, Uppsala, Sweden; China Medical University, TAIWAN

## Abstract

Disease spreads as a result of people moving and coming in contact with each other. Thus the mobility patterns of individuals are crucial in understanding disease dynamics. Here we study the impact of human mobility on HIV transmission in different parts of Kenya. We build an SIR metapopulation model that incorporates the different regions within the country. We parameterise the model using census data, HIV data and mobile phone data adopted to track human mobility. We found that movement between different regions appears to have a relatively small overall effect on the total increase in HIV cases in Kenya. However, the most important consequence of movement patterns was transmission of the disease from high infection to low prevalence areas. Mobility slightly increases HIV incidence rates in regions with initially low HIV prevalences and slightly decreases incidences in regions with initially high HIV prevalence. We discuss how regional HIV models could be used in public-health planning. This paper is a first attempt to model spread of HIV using mobile phone data, and we also discuss limitations to the approach.

## Introduction

Since the emergence of HIV/AIDS, modelling its dynamics has been dealt with extensively by many researchers. The basic approach is to describe the population in terms of three states: those who have not contracted HIV, *susceptible*, those who have HIV and remain sexually active, *infectious* and those individuals who are no longer engaged in spreading the disease, *removed*. This style of SIR model has been widely used to capture the dynamics of diseases, by for example [1–5] and in many other articles. For HIV, the SIR model is also applied, but with the assumption that infected individuals do not recover from the disease but rather stop being infectious [6–10]. SIR models consider the dynamics of diseases to depend only on the individual status like susceptible, infected or removed.

Under the standard SIR model, the transmission rates, infection periods, contact patterns and removed rates do not account for the spatial spread of the diseases. In this standard version, they ignore variations in demographic, social, cultural, economic and geographic factors [4, 11]. In reality, however, the spread of HIV is highly associated with geographic factors, such as population mobility, as well as the accessibility of and the proximity between infected areas [12]. Human mobility can be seasonal, short term and long term [13, 14]. Several studies have shown that migrant workers in urban areas can spread HIV to rural areas [15, 16]. For example, in South Africa, men who had moved away from a rural area to an urban area were twice as likely to have HIV than those still living in the rural area [12]. These migrant workers would regularly return to the rural area, potentially spreading the virus. Likewise, areas associated with high human mobility such as commercial farms and agricultural estates, mining areas, business centres and residential areas along busy roads have been connected to an increase in HIV infections [17–21]. Not all migrations appear to increase HIV transmission, and other studies have reported that the rural-urban migrations have little or no link to HIV risk [22, 23]. Overall, however, it appears that short-term human mobility, for example return visits from work to home town, are associated with high-risk behaviour. The migration to urban areas leads to an increased chance of interacting with individuals who are at higher-risk of being HIV infected, like sex workers [13, 14, 24]. Return visits to a home town then provide further spread of the disease.

Mathematical models that capture spatial scale for the spread of diseases dynamics are usually referred to as metapopulation models. In metapopulation models, the area under study is divided into different regions according to geographic positions. The distinct regions could be cities, towns or villages. The regions are connected by people travelling between them. Various studies have incorporated metapopulations in disease dynamics [25–30]. For example, Sattenspiel and Dietz [31], used a metapopulation model to show that increase in human mobility was associated with an increase of the spread of measles in the West Indian island of Dominica. Travel networks have very complex influence on disease dynamics, since they may fuel or may help disease extinction [29]. For example, Arino *et al* [32] used a two-region metapopulation model to study the influence of travel rates with respect to the stability of influenza. They observed that, for isolated regions, the disease in one of the regions approached the disease-free equilibrium while in the other region it approached the endemic. Introducing very small movements between the regions, the disease in all regions approached the endemic state. On increasing the movements rates, influenza died out in all the regions.

There have been a few earlier metapopulation models of the impact of human mobility on spread of HIV/AIDS. In one example, Coffee *et al* [13] looked at the impact of migration on the HIV epidemic in South Africa. They reported that, migrations when coupled to increased risk behaviour, have a causal effect on the increase of HIV. In a modelling study, Smith? *et al* [33] reported that, in order to globally eradicate HIV/AIDS, the money spent and other resources must aim at eradicating HIV/AIDS in all regions, since human mobility has an effect of sustaining the infections. However, the extent to which human mobility impacts the transmission of HIV infections still needs to be studied further. For example, one counterintuitive finding by Xiao *et al* [34], is that an increase in mobility rates among HIV/AIDS-infected individuals on mainland China decreased HIV infections in the country. Once again, it appears that there is a need to develop metapopulation models that are well-designed in order to clearly explain the impact of human mobility.

The purpose of this work is to develop a metapopulation model of the impact of human mobility to the transmission of HIV/AIDS. We modify the metapopulation model of Keeling and Rohani [35]. This model does not include a removed class, as is typical in HIV models. We thus introduce the removed class, which incorporates individuals who are in AIDS status and are no longer transmitting the disease. The model is parametrised using demographic data from Kenya at the time of the 2009 census [36] and the world bank data [37]. The rates of moving between regions is estimated from mobile phone data collected by Wesolowski *et al* [38]. Infection rate is estimated from Kenya HIV data KAIS 2007 [39] and the disease progression rate is adopted from the work of Baryama et al [9]. These data allow us to set up a full metapopulation HIV model for Kenya.

Our study builds on the work of Wesolowski *et al* [38], in analysing the impact of human mobility on malaria in Kenya. They used mobile phone data to track the regional travel of people between June 2008 and June 2009. Every call or message made by each user was mapped to one of 11,920 cell towers located in different regions in the country. From the call, an individual was assigned a primary settlement where they spent the majority of their night time. Using the primary settlements, they estimated the average monthly regional travel. We use their monthly mobility rate to study the impact of human mobility on HIV infections in Kenya.

## Methods

We now present a SIR metapopulation model for the dynamics of HIV/AIDS, following closely a model presented by Keeling and Rohani [35]. The adult population in region *i* is divided into susceptible *S*_*i*_, Infectious *I*_*i*_ and Removed *R*_*i*_, so that the total adult population in the region *i* is *N*_*i*_ = *S*_*i*_ + *I*_*i*_ + *R*_*i*_. The exposed period from time of HIV infection to a stage when infected individual becomes infectious is very short, so that when an individual acquires infections, immediately becomes infectious [40–43]. For this reason, we use the SIR model. The dynamics of each sub-population includes only individuals aged 15–64 years only, because this is the group of individuals who are sexually active, hence susceptible to HIV infections.

To include different regions in our model, we define *S*_*ij*_, *I*_*ij*_, *R*_*ij*_ and *N*_*ij*_ to be respectively the number of Susceptible, Infectious, Removed and total adult individuals currently visiting region *i* but who live in region *j*. For example, infected individuals working and spending most of their time in region *j* and going back to visit their family in region *i* are denoted *I*_*ij*_. We assume a homogeneous mixing of individuals within the regions, meaning that any infectious individual has the same probability of transmitting the disease to any susceptible individual in the population. We define *l*_*ji*_ to be the per individual rate per year of moving from region *i* to region *j*. We assume that *r* is the rate of return from visits to another region, which is assumed to be independent of the regions travelled between. We define $N_{i}=\sum_{j}N_{ij}=\sum_{j}S_{ij}+\sum_{j}I_{ij}+\sum_{j}R_{ij}=S_{i}+I_{i}+R_{i}$ to be the total adult population who are currently in region *i*.

We assume that the basic parameters governing the effects of the disease and population demographics are the same in all regions. We further assume that individuals return to their home region before departing for another region and there is no permanent migration and emigration between the sub-populations, so that individuals travel to other sub-population occasionally. In addition, we assume that the recruitment into classes occurs within home regions; i.e., *S*_*ii*_ is the only term that increases through population growth.

The susceptible class in region *i* are those newly-recruited into the sexually active cohort at the rate *ν*_*i*_ = *ν* × *p*_*i*_ individuals per year and the total susceptible individuals returning in region *i* from region *j* at the rate $\sum_{j}rS_{ji}$ individuals per year, where *ν* is the countrywide population growth rate and *p*_*i*_ is the proportion of the total adult population living in region *i*. Susceptibles are lost by becoming HIV infected at the rate $S_{ii}\beta_{i}\frac{\sum_{j}I_{ij}}{\sum_{j}N_{ij}}$, by dying of natural causes at the rate *μS*_*ii*_ and the total Susceptible individuals moving from region *i* to region *j* at a rate $\sum_{j}l_{ji}S_{ii}$ individuals per year respectively. Here *β*_*i*_ is the HIV transmission parameter or transmission probability for region *i*.

The equation describing the dynamics of the susceptible individuals *S*_*ii*_ in region *i* is given by

$$\begin{array}{c} \frac{dS_{ii}}{dt}=\nu_{i}-\beta_{i}S_{ii}\frac{\sum_{j}I_{ij}}{\sum_{j}N_{ij}}-S_{ii}\sum_{j}l_{ji}+\sum_{j}rS_{ji}-\mu S_{ii}. \end{array}$$ (1)

Susceptible individuals from region *j* who are current in region *i* are recruited by the rate which is governed by the number of people who move out from region *j*, at the rate *l*_*ij*_ *S*_*jj*_ and those returning to regional *j* at the rate *rS*_*ij*_ individuals per year respectively. The susceptible are lost by natural death at the rate *μS*_*ij*_ and those acquiring HIV infections at the rate $\beta_{i}S_{ij}\frac{\sum_{j}I_{ij}}{\sum_{j}N_{ij}}$ individuals per year respectively. Similarly, the equation describing the dynamics of the susceptible individuals *S*_*ij*_ in region *i* is given by

$$\begin{array}{c} \frac{dS_{ij}}{dt}=-\beta_{i}S_{ij}\frac{\sum_{j}I_{ij}}{\sum_{j}N_{ij}}+l_{ij}S_{jj}-rS_{ij}-\mu S_{ij}. \end{array}$$ (2)

The infectious population *I*_*ii*_ is recruited at a rate $\beta_{i}S_{ii}\frac{\sum_{j}I_{ij}}{\sum_{j}N_{ij}}$ and those returning at a rate $\sum_{j}rI_{ji}$ individuals per year respectively. Infectious are respectively reduced by those who die naturally at the rate *μI*_*ii*_, those who die due to the disease at the rate *δ*_*ii*_ *I*_*ii*_, those who progress to the removed class at the rate *γ*_*ii*_ *I*_*ii*_ and those travelling out the region at the rate $\sum_{j}l_{ji}I_{ii}$ individuals per year. Together this gives

$$\begin{array}{c} \frac{dI_{ii}}{dt}=\beta_{i}S_{ii}\frac{\sum_{j}I_{ij}}{\sum_{j}N_{ij}}-\gamma I_{ii}-I_{ii}\sum_{j}l_{ji}+\sum_{j}rI_{ji}-\mu I_{ii}-\delta I_{ii}. \end{array}$$ (3)

The infectious population *I*_*ij*_ is recruited by those acquiring HIV infections in region *i* at the rate $\beta_{i}S_{ij}\frac{\sum_{j}I_{ij}}{\sum_{j}N_{ij}}$ and from those coming for a visit at the rate *l*_*ij*_ *I*_*jj*_ individuals per year respectively. They are lost due to those who progress to the removed class at a rate *γI*_*ij*_, return to region *i* at a rate *rI*_*ij*_, die naturally at the rate *μI*_*ij*_ and die due to the disease at the rate *δI*_*ij*_ individuals per year respectively. The non-linear ordinary differential equation describing the dynamics of the infectious individuals *I*_*ij*_ in region *i* is given by

$$\begin{array}{c} \frac{dI_{ij}}{dt}=\beta_{i}S_{ij}\frac{\sum_{j}I_{ij}}{\sum_{j}N_{ij}}-\gamma I_{ij}+l_{ij}I_{jj}-rI_{ij}-\mu I_{ij}-\delta I_{ij}. \end{array}$$ (4)

The removed individuals *R*_*ii*_ are respectively recruited by infectious individuals *I*_*ii*_ who progress to the removed class at a rate *γI*_*ii*_ and those returning from visits from different regions a the rate *r*∑_*j*_ *R*_*ji*_ individuals per year. They are decreased due to those who visit other regions at the rate *l*_*ji*_ *R*_*ii*_, die due to the disease at the rate *δR*_*i*_ and die naturally at a rate *μR*_*i*_ individuals per year respectively. The non-linear ordinary differential equation describing the dynamics of the removed individuals *R*_*i*_ in region *i* is given by

$$\begin{array}{c} \frac{dR_{ii}}{dt}=\gamma I_{ii}-\sum_{j}l_{ji}R_{ii}+r\sum_{j}R_{ji}-\mu R_{ii}-\delta R_{ii}. \end{array}$$ (5)

The removed individuals *R*_*ij*_ are respectively recruited by infectious individuals *I*_*ij*_ who progress to the removed class at the rate *γI*_*ij*_ and those coming for a visit at the rate *l*_*ij*_ *R*_*jj*_ individuals per year. They are decreased due to those who return to region *j* at a rate *rR*_*ij*_, die due to the disease at the rate *δ*_*ii*_ *R*_*ij*_ and die naturally at a rate *μR*_*ij*_ individuals per year respectively. The non-linear ordinary differential equation describing the dynamics of the infectious individuals *R*_*ij*_ in region *i* is given by

$$\begin{array}{c} \frac{dR_{ij}}{dt}=\gamma I_{ij}+l_{ij}R_{jj}-rR_{ij}-\mu R_{ij}-\delta R_{ij}. \end{array}$$ (6)

**Fig 1:**
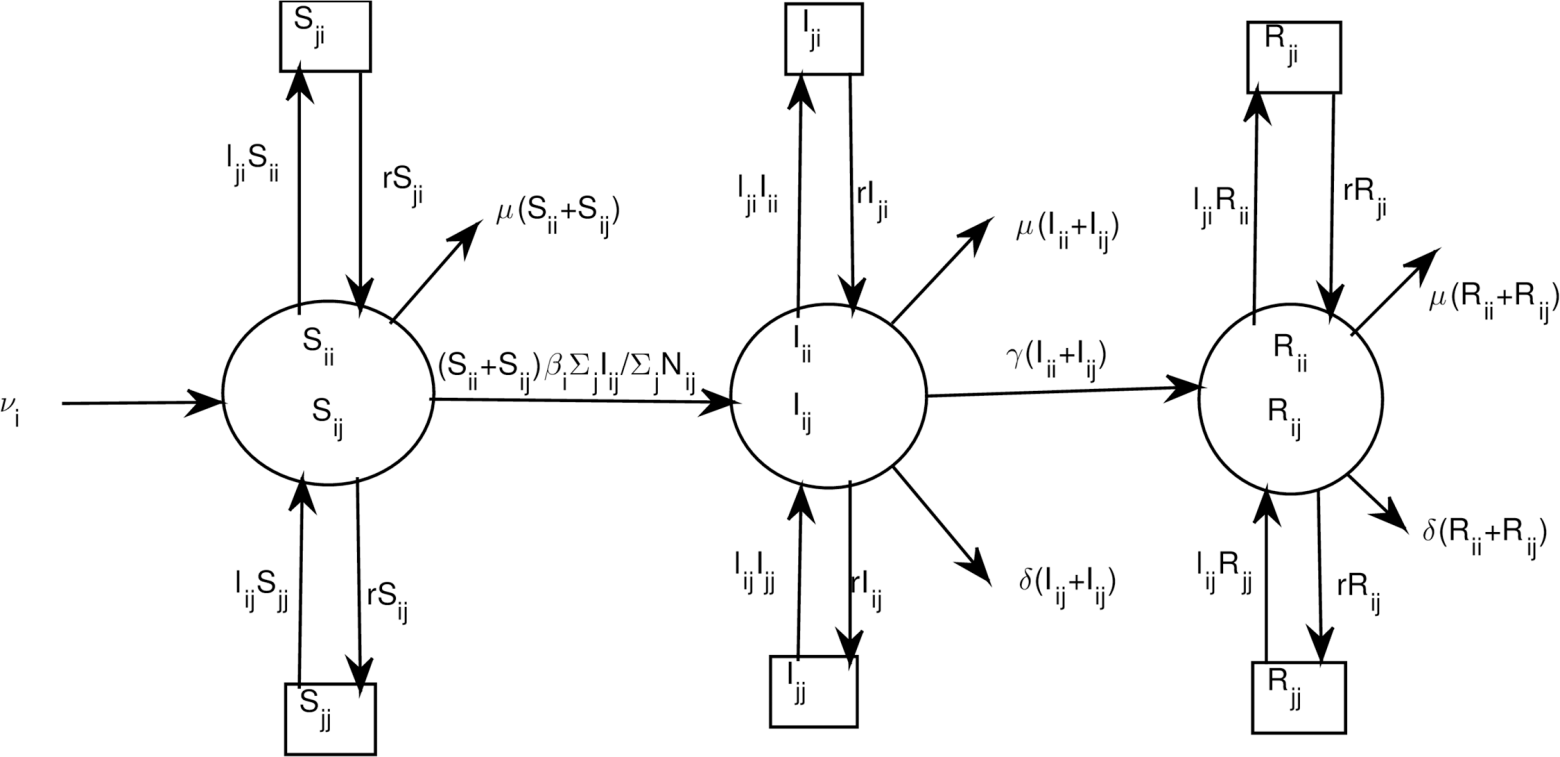
Flow diagram between regions for the SIR model with n-patches. For clarity, we consider individuals travelling to another region after returning home first.

The model is summarised in Fig 1.

To compute the basic reproduction number *R*_0_ of the system of equations from Eqs (1)–(6), we only consider equations of the states that include the infected individuals. These equations are referred to as the infected system [44]. In our model, we have assumed that the susceptible and the removed classes do not contribute to the transmission of HIV. The only class involved in the disease transmission is the infectious class. We therefore write the the system that represents the infectious individuals in a given region and those commuting between these regions as follows:

$$\begin{array}{ccc} \frac{dI_{ii}}{dt} & = & \beta_{i}S_{ii}\frac{\sum_{j}I_{ij}}{\sum_{j}N_{ij}}-\gamma_{ii}I_{ii}-\sum_{j}l_{ji}I_{ii}+\sum_{j}r_{ji}I_{ji}-\mu_{ii}I_{ii}-\delta_{ii}I_{ii},\\ \frac{dI_{ij}}{dt} & = & \beta_{i}S_{ij}\frac{\sum_{j}I_{ij}}{\sum_{j}N_{ij}}-\gamma_{ij}I_{ij}+l_{ij}I_{jj}-r_{ij}I_{ij}-\mu_{ij}I_{ij}-\delta_{ij}I_{ij},\\ \frac{dI_{ji}}{dt} & = & \beta_{j}S_{ji}\frac{\sum_{i}I_{ji}}{\sum_{i}N_{ji}}-\gamma_{ji}I_{ji}+l_{ji}I_{ii}-r_{ji}I_{ji}-\mu_{ji}I_{ji}-\delta_{ji}I_{ji},\\ \frac{dI_{jj}}{dt} & = & \beta_{j}S_{jj}\frac{\sum_{i}I_{ji}}{\sum_{i}N_{ji}}-\gamma_{jj}I_{jj}-\sum_{i}l_{ij}I_{jj}+\sum_{i}r_{ij}I_{ij}-\mu_{jj}I_{jj}-\delta_{jj}I_{jj}. \end{array}$$ (7)

The computation of *R*_0_ by the next-generation operator begins with equations of the system that involve the transmission part describing the production of new infections and then with those involve transition part, describing changes in state among the infected individuals [45].

The transmission part is represented by

$$\begin{array}{c} f_{i}=(\begin{array}{c} \beta_{i}S_{ii}\frac{\sum_{j}I_{ij}}{\sum_{j}N_{ij}}\\ \beta_{i}S_{ij}\frac{\sum_{j}I_{ij}}{\sum_{j}N_{ij}}\\ \beta_{j}S_{ji}\frac{\sum_{i}I_{ji}}{\sum_{i}N_{ji}}\\ \beta_{j}S_{jj}\frac{\sum_{i}I_{ji}}{\sum_{i}N_{ji}} \end{array}). \end{array}$$

$$\begin{array}{c} F=\frac{\partial f_{i}}{\partial X}(DFE)=[\begin{array}{cccccccccc} \frac{\beta_{i}S_{ii}^{*}}{\sum_{j=1}^{n}N_{ij}} & . & . & . & \frac{\beta_{i}S_{ii}^{*}}{\sum_{j=1}^{n}N_{ij}} & 0 & 0 & 0 & 0 & 0\\ \frac{\beta_{i}S_{ij}^{*}}{\sum_{j=1}^{n}N_{ij}} & . & . & . & \frac{\beta_{i}S_{ij}^{*}}{\sum_{j=1}^{n}N_{ij}} & 0 & 0 & 0 & 0 & 0\\ . & . & . & . & . & 0 & 0 & 0 & 0 & 0\\ . & . & . & . & . & 0 & 0 & 0 & 0 & 0\\ . & . & . & . & . & 0 & 0 & 0 & 0 & 0\\ \frac{\beta_{i}S_{in}^{*}}{\sum_{j=1}^{n}N_{ij}} & . & . & . & \frac{\beta_{i}S_{in}^{*}}{\sum_{j=1}^{n}N_{ij}} & 0 & 0 & 0 & 0 & 0\\ 0 & 0 & 0 & 0 & 0 & \frac{\beta_{j}S_{ji}^{*}}{\sum_{i=1}^{n}N_{ji}} & . & . & . & \frac{\beta_{j}S_{ji}^{*}}{\sum_{i=1}^{n}N_{ji}}\\ 0 & 0 & 0 & 0 & 0 & . & . & . & . & .\\ 0 & 0 & 0 & 0 & 0 & . & . & . & . & .\\ 0 & 0 & 0 & 0 & 0 & . & . & . & . & .\\ 0 & 0 & 0 & 0 & 0 & \frac{\beta_{j}S_{jn}^{*}}{\sum_{i=1}^{n}N_{ji}} & . & . & . & \frac{\beta_{j}S_{jn}^{*}}{\sum_{i=1}^{n}N_{ji}}\\ 0 & 0 & 0 & 0 & 0 & \frac{\beta_{j}S_{jj}^{*}}{\sum_{i=1}^{n}N_{ji}} & . & . & . & \frac{\beta_{j}S_{jj}^{*}}{\sum_{i=1}^{n}N_{ji}} \end{array}]. \end{array}$$

Here S* represents the susceptible population at a disease free equilibrium point. Therefore *F* = *F*_1_ ⊕ *F*_2_ ⊕ … ⊕ *F*_*n*_.

The transition part, describing changes in state, is obtained from

$$\begin{array}{c} v_{i}=(\begin{array}{c} \gamma_{ii}I_{ii}+\sum_{j=1}^{n}l_{ji}I_{ii}-\sum_{j=1}^{n}r_{ji}I_{ji}+\mu_{ii}I_{ii}+\delta_{ii}I_{ii}\\ \gamma_{ij}I_{ij}-l_{ij}I_{jj}+r_{ij}I_{ij}+\mu_{ij}I_{ij}+\delta_{ij}I_{ij}\\ \gamma_{ji}I_{ji}-l_{ji}I_{ii}+r_{ji}I_{ji}+\mu_{ji}I_{ji}+\delta_{ji}I_{ji}\\ \gamma_{jj}I_{jj}+\sum_{i=1}^{n}l_{ij}I_{jj}-\sum_{i=1}^{n}r_{ij}I_{ij}+\mu_{jj}I_{jj}+\delta_{jj}I_{jj} \end{array}). \end{array}$$

$$\begin{array}{c} V=\frac{\partial v_{i}}{\partial X}(DFE)=[\begin{array}{cccccccccc} \kappa_{ii}+\sum_{j=1}^{n}l_{ji} & 0 & 0 & -r_{ji} & . & . & 0 & -r_{ni} & 0 & 0\\ 0 & \kappa_{ij}+r_{ij} & 0 & 0 & . & . & 0 & 0 & 0 & -l_{ij}\\ . & . & . & . & . & . & . & . & . & .\\ . & . & . & . & . & . & . & . & . & .\\ . & . & . & . & . & . & . & . & . & .\\ 0 & 0 & \kappa_{in}+r_{in} & 0 & . & & 0 & 0 & 0 & -l_{in}\\ -l_{ji} & 0 & 0 & \kappa_{ji}+r_{ji} & . & . & 0 & 0 & 0 & 0\\ . & . & . & . & . & . & . & . & . & .\\ . & . & . & . & . & . & . & . & . & .\\ . & . & . & . & . & . & . & . & . & .\\ -l_{jn} & 0 & 0 & 0 & . & . & 0 & 0 & \kappa_{jn}+r_{jn} & 0\\ 0 & 0 & -r_{ij} & 0 & . & . & -r_{nj} & 0 & 0 & \kappa_{jj}+\sum_{i=1}^{n}l_{ij} \end{array}]. \end{array}$$

Here *κ*_*ii*_ = *γ*_*ii*_ + *μ*_*ii*_ + *δ*_*ii*_, *κ*_*ij*_ = *γ*_*ij*_ + *μ*_*ij*_ + *δ*_*ij*_, *κ*_*in*_ = *γ*_*in*_ + *μ*_*in*_ + *δ*_*in*_, *κ*_*ji*_ = *γ*_*ji*_ + *μ*_*ji*_ + *δ*_*ji*_, *κ*_*jn*_ = *γ*_*jn*_ + *μ*_*jn*_ + *δ*_*jn*_, and *κ*_*jj*_ = *γ*_*jj*_ + *μ*_*jj*_ + *δ*_*jj*_.

**Theorem 1**: Let $A=\left[a_{ij}\right]\in R^{n\times n}$ have *a*_*ij*_ ≤ 0 for *i* ≠ *j*. If the sum of the entries in each column is positive, then *A* is non-singular m-matrix [46, 47].

By Theorem 1, it can be observed that the matrix *V* is a non-singular m-matrix; hence *V* ^−1^ exists.

The basic reproduction number *R*_0_ as proposed by van den Driesche and Whatmough [45], is the the spectral radius of the matrix *G* = *FV* ^−1^ = (*F*_1_⊕…⊕*F*_*n*_)*V* ^−1^. So that *R*_0_ = max{|*λ*_1_, *λ*_2_, …*λ*_*k*_|, *λ*_1_, *λ*_2_, …*λ*_*k*_ ∈ *σ*(*G*)} = *σ*(*G*).

In this case, the basic reproduction numbers can not be written explicitly. However, it can be observed that the basic reproduction number depends on the travel rates, demographic and the epidemic parameters. Therefore, given the set of parameter values and the travel rates, *R*_0_ can be computed numerically.

Note that, in the absence of mobility and regions (with *β*_*i*_ = *β*, *l*_*ij*_ = 0 and *r* = 0), the model is reduced to the standard SIR model

$$\begin{array}{ccc} \frac{dS}{dt} & = & \nu -\beta S\frac{I}{N}-\mu S,\\ \frac{dI}{dt} & = & \beta S\frac{I}{N}-(\gamma +\mu +\delta )I,\\ \frac{dR}{dt} & = & \gamma I-(\mu +\delta )R,\\ \frac{dN}{dt} & = & \nu -\mu N-\delta I-\delta R. \end{array}$$ (8)

which has $R_{0}=\frac{\beta }{\delta +\mu +\gamma }$ as the basic reproduction number.

## Model Parameters

We start with the disease-related parameters, which are assumed to be the same for all regions. The progression rate from HIV to AIDS denoted by *γ* is 0.06897 < *γ* < 0.3846; this is because the HIV incubation period is between 2.6 to 14.5 years; [48]. The life expectancy in the AIDS class is about 8 years, so the HIV/AIDS-induced death rate *δ* is ≈0.125 [9]. The average life expectancy at birth of Kenya from 1960 to 2011 is about 57.9 years (see S1 Table), so the natural death rate *μ* is approximated to be 0.01727 per year and the average growth rate *ν* of Kenya from 1960 to 2011 is estimated to be 0.031648 [37].

We then look at the population and movement parameters. S2 Table gives the adult population for 2009 [36] and the estimate of people infected with HIV in 20 regions of Kenya for 2007 [39]. The rate at which individuals from region *j* visit region *i*, denoted by *l*_*ij*_ individuals per year, is estimated from the monthly average number of trips per 1000 individuals over the course of the year (see S3 Table). The data is then divided by 1000 to get the average number of trips for an individual per year. We assume that visits last on average one week, so *r* = 52. Mobile phone data does not allow us to estimate this parameter more accurately, so we take this estimate as a starting point.

A challenge is to estimate the spread of the infection in various regions. In the absence of regional variation, this can be estimated from the basic reproduction number *R*_0_. Specifically, if we assume that Eqs (1)–(6) have no regions and movement terms (i.e. *l*_*ij*_ = *r* = 0) we then have *β*_*i*_ = *β* and

$$\begin{array}{c} \beta =R_{0}(\gamma +\delta +\mu ). \end{array}$$

The estimates of the HIV basic reproduction number *R*_0_ worldwide is not clearly set. However, the available estimates varies much from country to country. In some countries, their estimates are very small and are even below one. For example, the HIV basic reproduction number for men having sex with men in Denmark is reported to be between 0.44 and 0.6, in Norway it is between 0.57 and 1.27, and in Sweden it is between 0.36 and 0.95 [49]. But in other countries, the estimates are above one. For example, in West Germany, the HIV *R*_0_ is reported to lie between 3.43 and 4.08, in France it is between 3.38 and 3.381, and in the UK it is between 3.38 and 3.96 [50]. At the moment, we do not have good estimates of the basic reproduction number of HIV in Kenya. However, a study by Williams and Gouws [51] has reported that the *R*_0_ for HIV for some African countries ranges between 1.94 and 8.93, and Kenya having 6.3 as an estimate of the HIV *R*_0_ and the median value being 4.6 for sub-Saharan African countries. This shows that much work is needed to decrease the basic reproduction number below one.

The absence of good estimates for *R*_0_ for both regions and Kenya as a whole limits the analysis we can do. However, we are interested in the role of space in determining the spread of the disease and to account for differences between regions (i.e. *β*_*i*_ ≠ *β*). We therefore investigate two different scenarios, one in which *R*_0_ for Kenya as a whole is slightly below one, and the other where *R*_0_ is slightly greater than one. The aim of looking at these two scenarios is to get an overall understanding of the role of how regional (HIV/AIDS) differences influence the spread of disease under these scenarios, rather than to give exact predictions of how many people will have HIV in the future.

With this background, we set a parameter *R*_*B*_ that gives the baseline *R*_0_ for Kenya. We then set the *β*_*i*_ for the individual regions to be

$$\begin{array}{c} \beta_{i}=R_{B}\left(\gamma +\delta +\mu \right)\exp(\frac{I_{i}\left(0\right)}{H}). \end{array}$$

where *I*_*i*_(0) is the initial infectious individuals in region *i* and *H* is chosen to be the population mean of the most populated region, and in this case Nairobi is the most populated region as shown in S4 Table. The idea here is that those regions that currently have higher levels of infection have a higher value of disease transmission *β*_*i*_. The choice of *H* as a rate conversion constant is arbitrary, but it ensures that the *β*_*i*_’s do not become too large.

**Fig 2:**
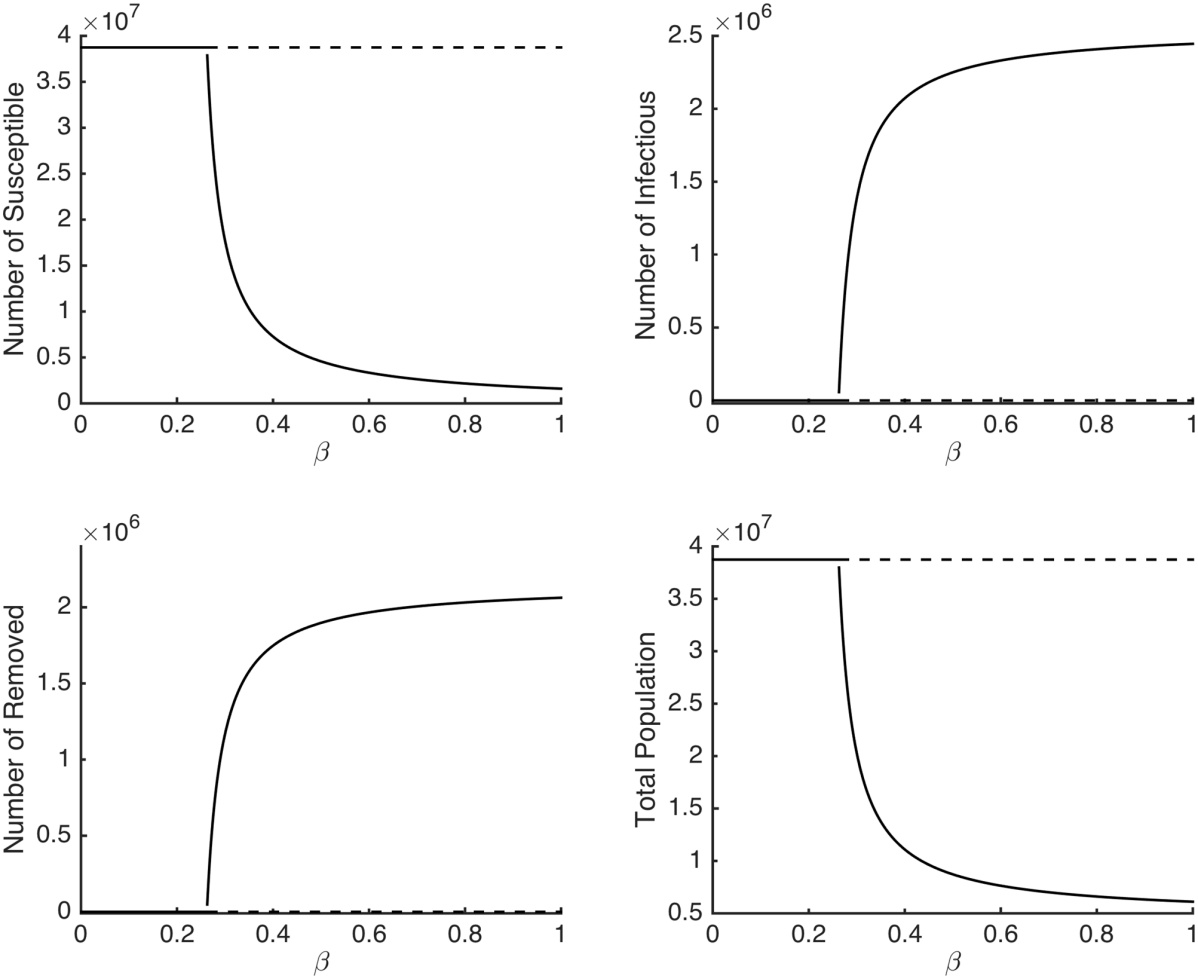
Bifurcation diagram of the of the system in Eq (8). The solid and dotted lines show the values at which the disease-free equilibrium point is stable and unstable respectively. The solid curves show the values at which the endemic equilibrium point is stable.

In order to set the baseline *R*_*B*_ for Kenya from the metapopulation model, we plot the bifurcation diagram for the SIR model in the absence of movements (i.e., Eq (8)). From Fig 2, we remark that *β* ≈ 0.2623 is the threshold value for the existence or nonexistence of the disease for all regions. Choosing *β* = 0.22 gives *R*_0_ = 0.8388 and choosing *β* = 0.42 gives *R*_0_ = 1.6014. We therefore set the *R*_*B*_ = 0.8388 for *R*_0_ < 1 and *R*_*B*_ = 1.6014 for *R*_0_ > 1.

**Fig 3:**
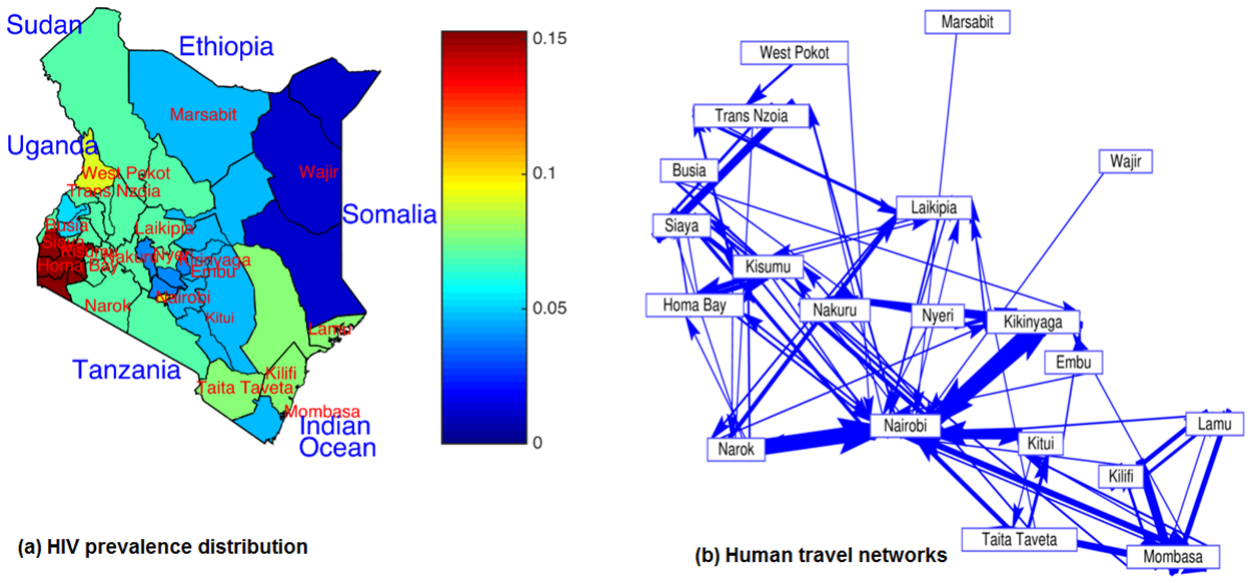
(a) Map of Kenya showing HIV prevalence distributions. The color bar from blue to red is in the order of increasing HIV prevalence. For clarity, the names of counties included are the only ones included in this study (Source of data:ArcGIS.com: shapefile-The 47 counties of Kenya (shapefile by dmuthami S5 Table) and HIV data from [39]. (b) Human travel networks (S6 Table) as estimated by [38]. Monthly average number of trips per 1000 individuals between all pairs of regions over the course of the year. For clarity, only trips made per 1000 individuals that are more than 60 trips per year are shown, with arrows indicating the direction of movements from home region to a visited region. The thickness of the arrow represents the number of trips made.

The prevalence of HIV infections in Kenya is distributed as shown in Fig 3a. Nyanza region, which includes Homa Bay, Kisumu and Siaya, has the highest percentage (15.5%); followed by Nairobi with 9.0%; coast region, which includes Lamu, Mombasa, Taita Taveta and Kilifi with 7.9%; rift valley, which includes West Pokot, Laikipia, Trans Nzoia, Narok and Nakuru with 7.0%; western region, which includes Kakamega, Busia with 5.1%; eastern region, which includes Marsabit, Embu, and Kitui with 4.7%; central region, which includes Nyeri and Kilinyaga with 3.8%; and the north Eastern region, which includes Wajir with the lowest adult HIV prevalences of 1.0% (S4 Table).

In Fig 3b, we see that Nairobi is the main hub of the movements of people from all regions in Kenya. We also observe that the regions that are less connected seem to have low HIV prevalence rates. For example, Wajir region has 1.0% of HIV prevalence and is the list-connected region.

## Results

**Fig 4:**
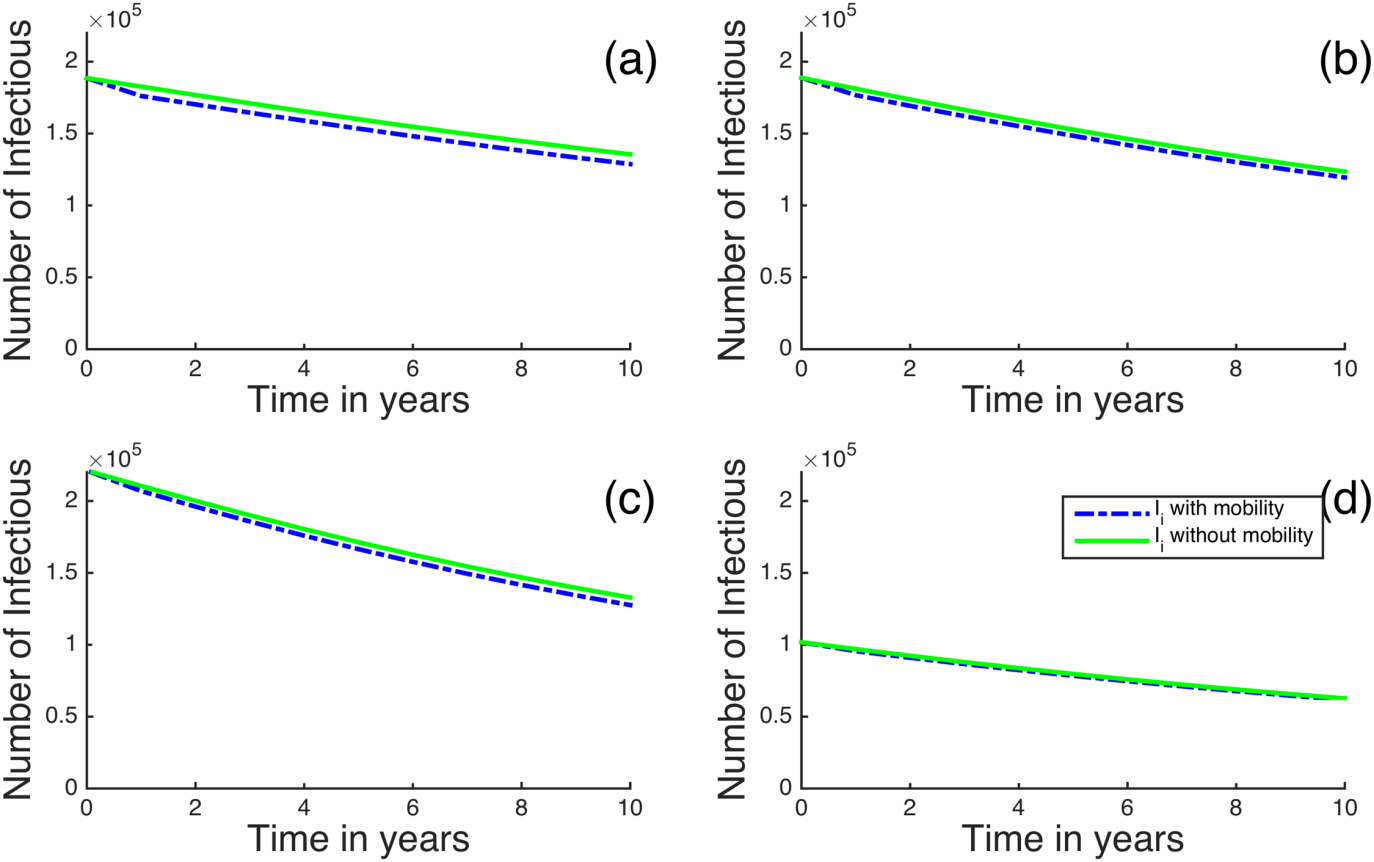
Time evolution of the metapopulation model for (a) Nairobi, (b) Kisumu, (c) Homa Bay and (d) Siaya for *R*_*B*_ < 1. The dotted blue lines represent infectious individuals when there is human mobility and the solid green lines represent infectious individuals when there is no human mobility between the regions.

We first look at our model for the four regions in Kenya with the highest HIV/AIDS prevalences: Nairobi, Kisumu, Homa Bay and Siaya [39]. Fig 4 shows the case where *R*_*B*_ < 1 so that the disease is decreasing overall. When human mobility is included, the infectious and the removed individuals decrease even more rapidly than in the case where no mobility is included in the model. Homa Bay has a higher percentage of infectious individuals compared to the other regions, followed by Nairobi and Kisumu. For regions with higher prevalence, the mobility has a observable but relatively small effect on the disease dynamics. For example, after 8 years if mobility is not included Siaya region has 68,910 infectious individuals, while including mobility it has 67,790 infectious individuals. This is approximately a 1.6% decrease.

**Fig 5:**
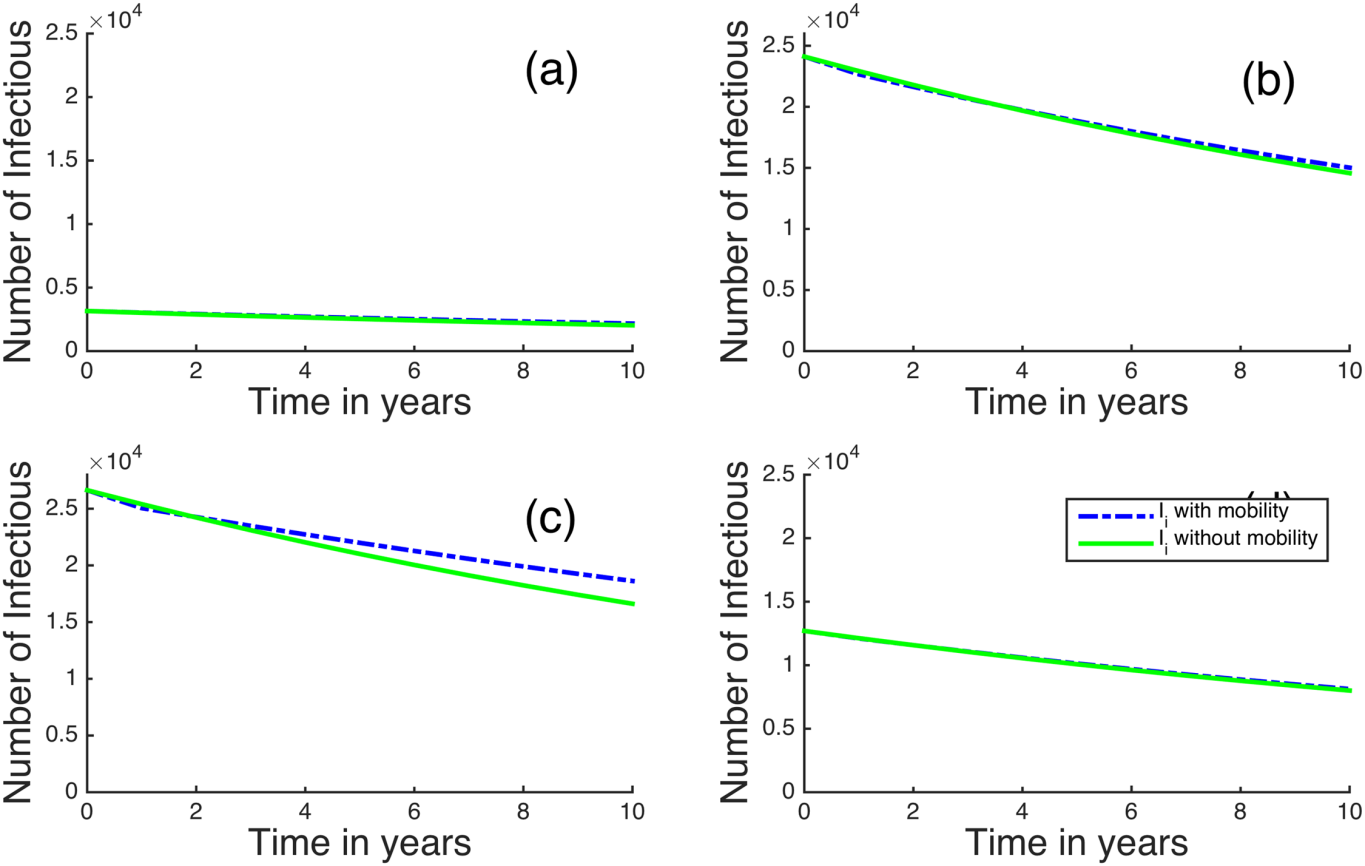
Time evolution of the metapopulation model for (a) Wajir, (b) Laikipia, (c) Kirinyaga, (d) Marsabit for *R*_*B*_ < 1. The dotted blue lines represent infectious individuals when there is human mobility and the solid green lines represent infectious individuals when there is no human mobility between the regions.

In Fig 5, we plot the results when *R*_*B*_ < 1 for the four regions with the smallest initial HIV/AIDS prevalences: Wajir, Laikipia, Kirinyaga and Marsabit [39]. For these regions, including human mobility in the model dynamics slightly increases the number of infectious individuals in all the selected regions. For example, after 8 years without human mobility, Wajir region has 2,217 infectious individuals, while including human mobility gives 2,334 infectious individuals. This is approximately a 5% increase. It seems that human mobility tends to increase infectious individuals in regions with initially low HIV prevalences.

**Fig 6:**
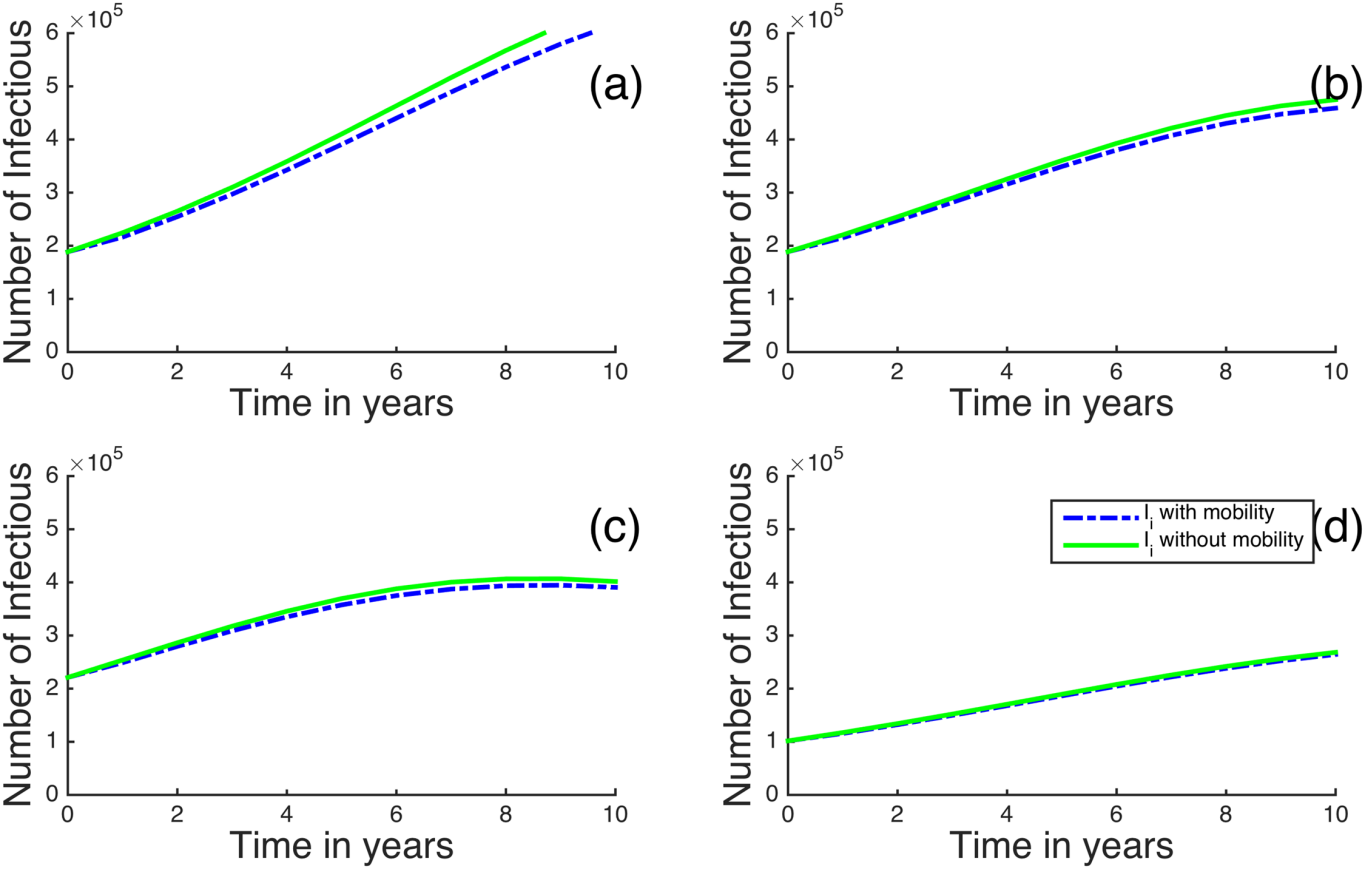
Time evolution of the metapopulation model for (a) Nairobi, (b) Kisumu, (c) Homa Bay and (d) Siaya for *R*_*B*_ > 1. The dotted blue represent infectious individuals when there is human mobility and the solid green lines represent Infectious individuals when there is no human mobility between the regions.

**Fig 7:**
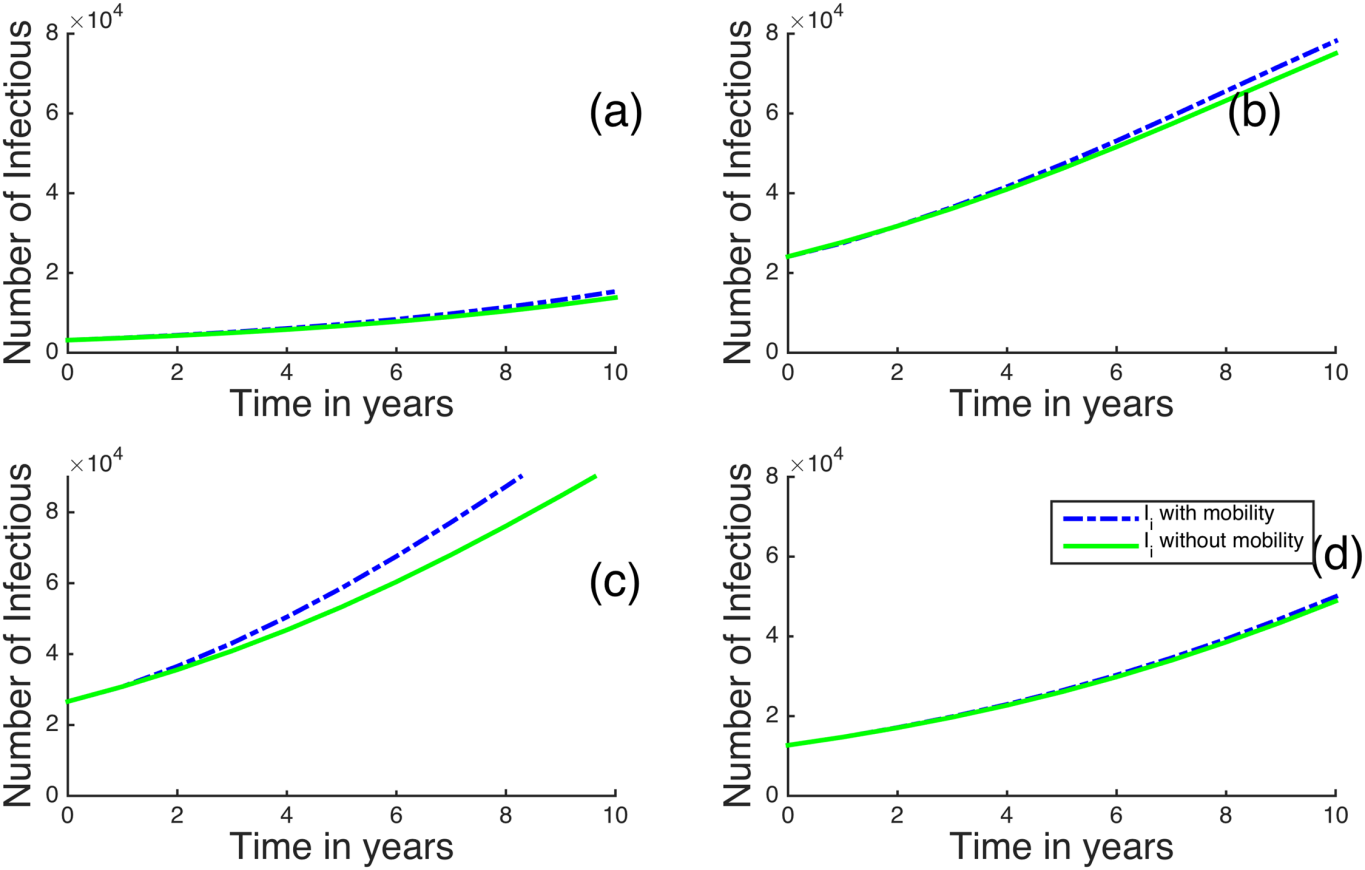
Time evolution of the metapopulation model for (a) Wajir, (b) Laikipia, (c) Kirinyaga, (d) Marsabit for *R*_*B*_ > 1. The dotted blue lines represent infectious individuals when there is human mobility and the solid green lines represent Infectious individuals when there is no human mobility between the regions.

In Fig 6, we plot the results for the regions with the highest HIV prevalence when *R*_*B*_ > 1. Once again, it seems that human mobility tends to slightly decrease the number of infectious individuals in these regions. For example, if the mobility is not included between the regions, after 8 years we see that Nairobi has an 567,500 infectious individuals, but if we introduce mobility in the model, it has an approximate of 536,300 infectious individuals; this is almost a 5.5% decrease. Fig 7 shows the dynamics of the four regions with the lowest initial HIV prevalences. It can also be observed that human mobility tends to increase the infectious individuals in regions with initially low HIV prevalences. For example, after 8 years without mobility, Marsabit region has 38,550 infectious individuals, while including human mobility gives 39,320 infectious individuals; this is approximately a 2% increase.

Based on the results for the regions above, we see that human mobility decreases new HIV infections in the regions whose initial HIV prevalence is high and it increases new HIV infections in regions with low HIV prevalence. To summarise the effect of infections across regions, we look at the incidence rate. We define the incidence rate as estimated from the log of the gradient of the growth of the infectious class [52, 53]. First we find the increases of *I*_*i*_ for each region. The solutions follow exponential growth, and we thus transform the solutions by taking the log. The region with the biggest positive slope corresponds to the region with highest increase in incidence rate of HIV infectious. The region with biggest negative slope corresponds to the one with the biggest decrease in incidence rate of the HIV infections.

**Fig 8:**
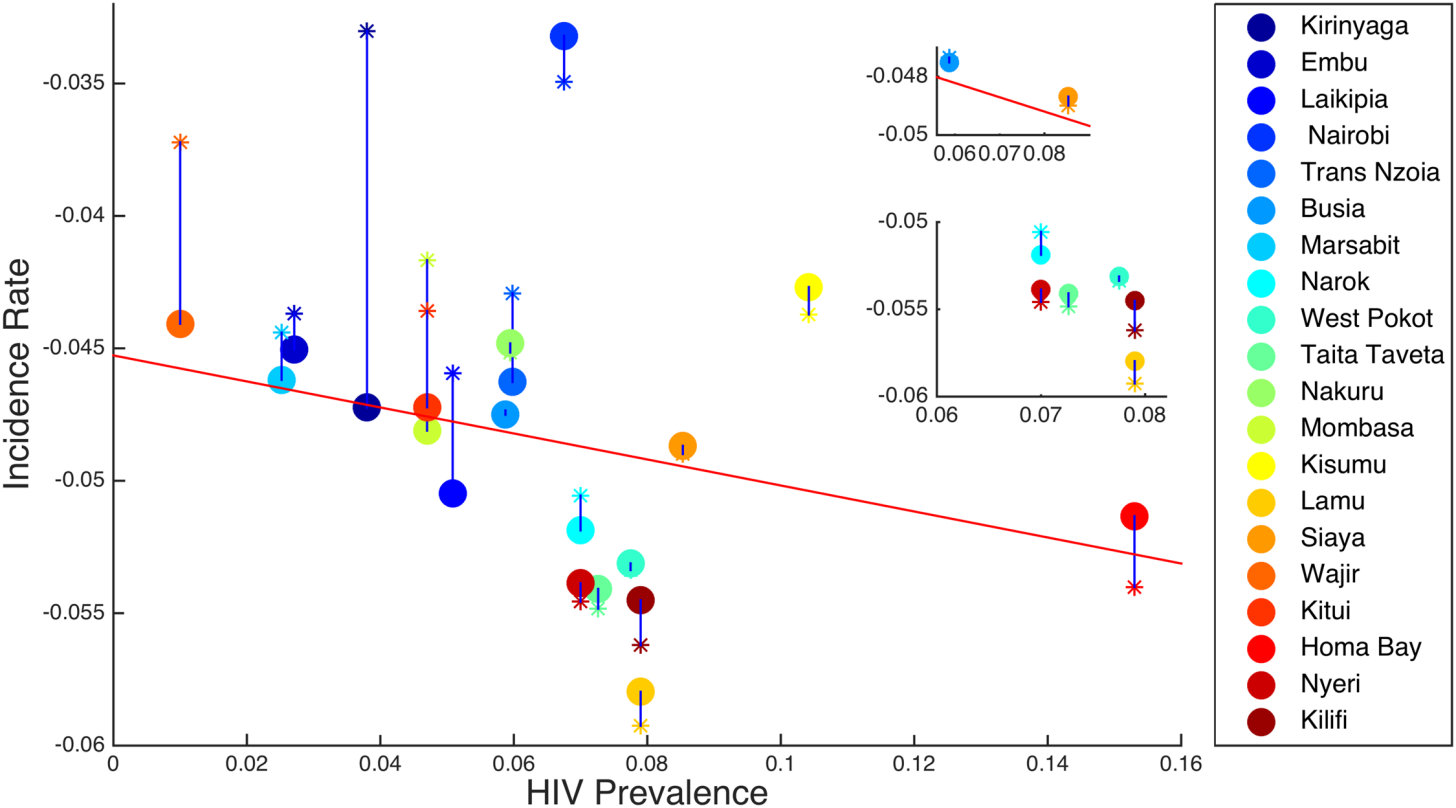
Incidence rate versus prevalence rate. A dot represents the dynamics of HIV infections without human mobility between the regions and a star represents the dynamics of HIV infections when there is human mobility between the regions for *R*_*B*_ < 1.

In Fig 8, we plot the incidence rate against the HIV prevalence rate, when there is no mobility and when there is mobility for *R*_*B*_ < 1. It can be seen that human mobility tends to increase the HIV incidences for the regions with initially low HIV prevalence; at the same time it tends to decrease the incidence rate of most of the regions with initially high HIV prevalence. Specifically, with the exception of Narok and Nairobi, it can also be observed that 7% is a threshold value of initial HIV prevalence above which human mobility decreases the HIV infection rate; below this value, human mobility tend to increase the HIV infections rate in a region.

**Fig 9:**
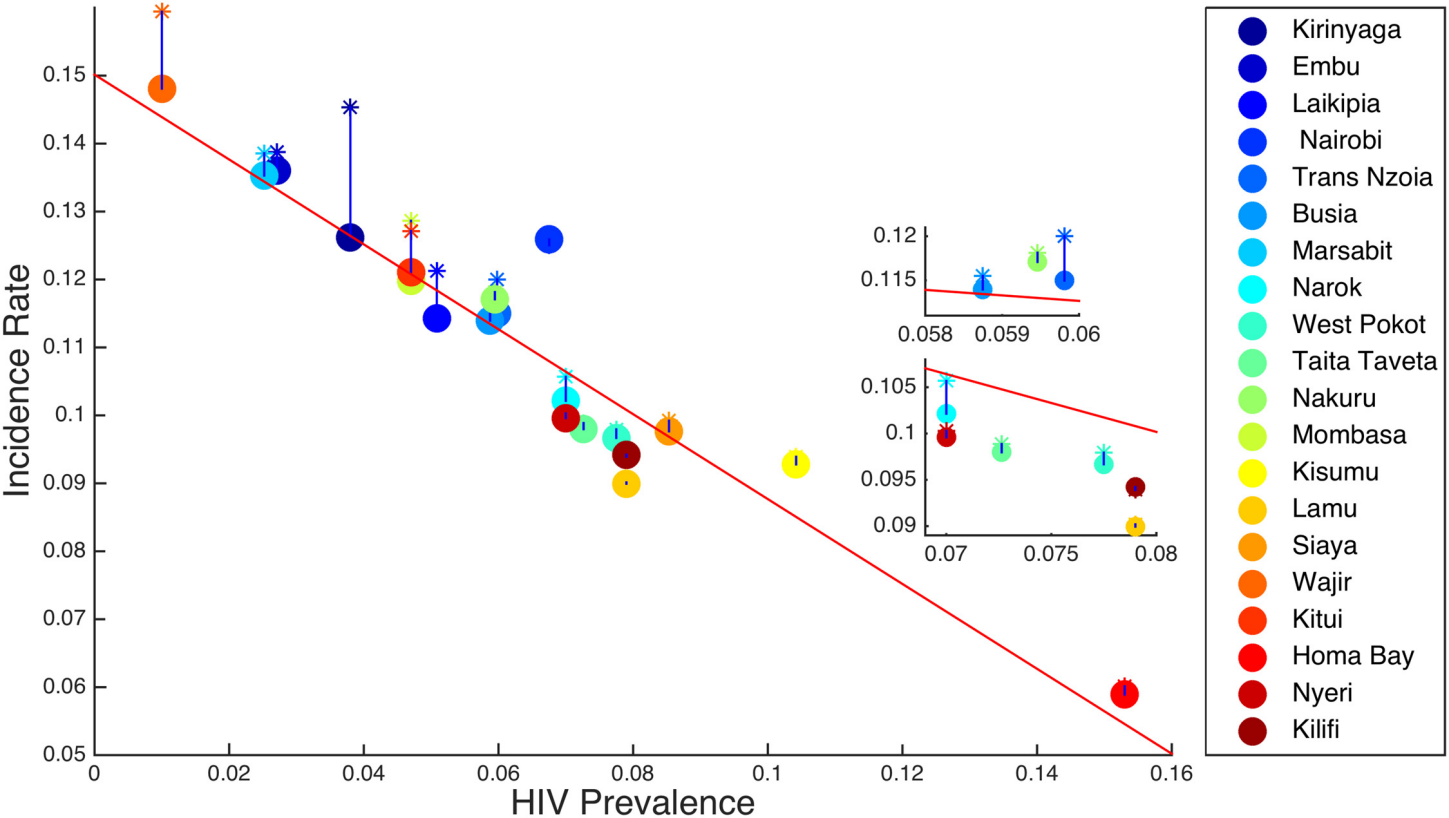
Incidence rate versus prevalence rate. A dot represents the dynamics of HIV infections without human mobility between the regions and a star represents the dynamics of HIV infections when there is human mobility between the regions for *R*_*B*_ > 1.

In Fig 9, we plot the incidence rate against the HIV prevalence rate for *R*_*B*_ > 1. In this case, mobility increases the incidence rates in regions with low HIV prevalence and also slightly increases the incidence rates in the regions with high HIV prevalences, but the increase is very small compared to regions with low HIV prevalences.

**Fig 10:**
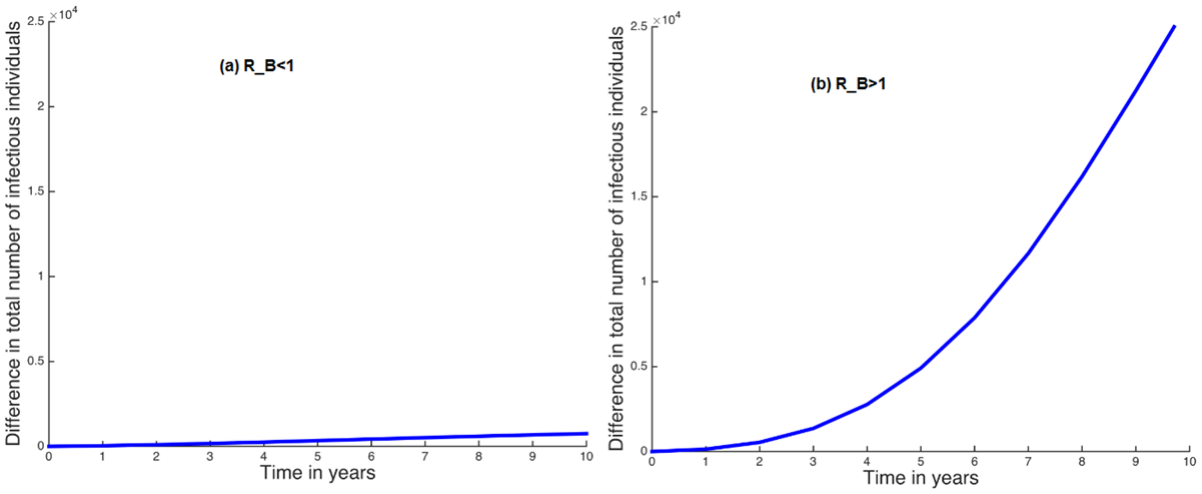
(a) and (b) show the differences between the total number of infectious individuals when there is human mobility and when there is no human mobility within the regions in the country for *R*_*B*_ < 1 and *R*_*B*_ > 1 respectively.

We also look at the country-wide impact of human mobility on HIV infections. In Fig 10a, we plot the difference of the total number of infectious in the country when *R*_*B*_ < 1. Mobility slightly increases the number of infectious individuals in the country as a whole. For example, without including human mobility between the regions, after 8 years Kenya would have 902,300 infectious individuals, while including human mobility between its regions it has 600 additional infectious individuals. This is only a 0.07% increase. For *R*_*B*_ > 1, we observe that human mobility increases the overall number of infectious individuals in the country (Fig 10b). We observe that, without human mobility in this case, Kenya would have 3,232,000 infectious individual compared to 3,248,000 infectious individuals with mobility. This gives a difference of 16,000 infectious individuals, an approximately 0.5% increase.

## Discussion

Our results show, using countrywide data, that human mobility can influence to the transmission of HIV. However, mobility plays a relatively small role. For example, we estimate that, over an eight year period in Kenya as a whole, it can contribute to about 0.5% additional cases of HIV. This contribution is even smaller if the disease decreases overall in the population. These results contrast with some other studies that report a positive correlations between mobility and the HIV prevalence [19, 54]. For example, a study of east and south Africa long-distance truck drivers found that mobility is the main contributing factor to the HIV transmission [55–58]. When a disease first spreads, we also expect mobility to play an important role. For example, air-transportation networks have been responsible for the global spread of severe acute respiratory syndrome (SARS) [59], and, because of the higher mobility in Europe, pandemic influenza could diffuse rapidly [60]. In our study, in which HIV is already endemic, overall mobility appears to make only a small difference to the total spread of the disease in Kenya.

Mobility does play an important role in local dynamics of HIV. Specifically, regions with initially low HIV prevalences experience an increase in the number of infectious individuals when mobility is accounted for, while regions with initially high HIV prevalences experience a slight decrease. Even when the overall trend is for the disease to decrease (i.e *R*_*B*_ < 1) human mobility tends to delay eradication in low prevalence regions, and slightly speed up the eradication process in regions with high HIV prevalences. When the disease is in an endemic state, regions with initially low HIV prevalences tend to increase their HIV incidence rates more than the regions with initially high HIV prevalences. Here our results seem to be in agreement with other studies, which report that migrant workers in urban areas can spread HIV to rural areas on their trips back to their areas of origin [12, 15, 16]. For example, if we compare big cities, like Nairobi, and small cities, like Kirinyanga, mobility tends to slightly decrease the number of infectious individuals in the former and slightly increase infections in the latter. Infectious individuals travel from the most infected, typically urban areas, and infect susceptibles in the least infected, rural areas.

There are also differences between different high prevalence regions. For example, in Nairobi, initially infections grow faster than other regions, irrespective of mobility (Fig 6). When *R*_*B*_ < 1, Nairobi converges very slowly to the disease-free equilibrium point compared to other regions (Fig 4). This can be attributed to the fact that Nairobi is the most populated region in the country and highly connected to other regions (Fig 3b). This result concurs with other studies, which associates high HIV infections with high human mobility in areas such as commercial farms and agricultural estates, mining areas, business centres and residential areas along busy roads [17–21]. It seems that more infectious individuals move from the most infected areas like Homa Bay, Siaya, Kisumu and other regions to Nairobi.

Our study suggests some policies for controlling HIV. For example, policies should concentrate on educating people working in urban areas to take care in their sexual relations while in the urban area by promoting safe sex, encouraging condom use, reducing the number of sex partners or reducing risky behaviours like having sex with sex workers [12, 15, 61]. Potentially, if migrants could travel with their partners to urban areas [14], then this would reduce the growth of the disease in rural areas. Proper education aimed at promoting behaviour changes and avoiding risk behaviours is crucial [61, 62].

Several improvements to our model are needed if we wanted to make quantitative predictions about HIV spread across regions. First, we did not account for state-dependent movements, where *l*_*ij*_ differs for infectious and susceptibles. For example, there is stigmatization and discrimination of people living with HIV, so infected individuals tend to move away or hide their HIV status after being diagnosed [63, 64]. Secondly, we did not consider intervention in our model, through education strategies for example, which could lead to behaviour changes of susceptible and infectious individuals. Similarly, we do not model precautions taken by individuals who might avoid having sexual relationships with people coming from the highly infected areas [61, 62]. Including preventative measures would reduce the susceptible populations and hence decrease new infections. Finally, we treat those individuals who travel as being no more likely to engage in risky sexual behaviour than other people in the regions they live. Given that many of the visits from urban to rural areas are made by people who might exhibit more risky behaviour when living alone in the city [13, 14, 24], our original assumptions may not be justified.

The human mobility data we used were obtained from mobile phones. Such data can only be collected in areas where there are cell phone towers; as a result, cross-border migrants were not captured [38, 65]. Mobile phone data tends to under estimate the number of between-area visits and can lead to incorrect estimates in the number of clinical outcomes compared to official census data [66]. For example, in Kenya, in the urbanised area, malaria transmission estimated from mobile data were higher than the clinical cases, while in the peripheral areas, malaria clinical cases were higher than those estimated from mobile phone data [38]. Mobile phone data should be used as a substitute in the case of unavailability of higher-resolution data and as a useful starting point [66].

In our case, these data have helped us analyse the impact of human mobility on the transmission of HIV in Kenya. This provides a first metapopulation model of how human mobility can influence HIV/AIDS infections within a country based on the region’s initial HIV prevalence and can be improved on as data becomes available. Despite the minor impact of mobility observed in this study, mobility should not be underestimated and neglected. We suggest that the health practitioners and policy makers incorporate the impact of human mobility in the HIV/AIDS transmission-control programmes.

## Supporting Information

S1 Table This file contains the life expectancy, the percentage of adult population aged (15–64) years old in Kenya from 1960–2011. It also provides the calculated average growth rate and the death rate of the country for 54 years.
(CSV)

S2 Table This file provides the number of adult population, the number of susceptible, the number of infectious and the number of removed individuals for twenty combined regions. (PDF)

S3 Table This file gives the monthly average number of trips (return rate) per individuals between all pairs of 20 regions over the course of the year. (PDF)

S4 Table This file provides the 47 counties of Kenya and their respective population size for 2009 and the HIV prevalence for 2007. (PDF)

S5 Table This file gives the shape-files data of the 47 counties of Kenya. (CSV)

S6 Table This file provides the average number of trips per month between all pairs of 20 regions. (PDF)

S1 Figs This file gives the list of all figures used in this work. (PDF)
