# Supplementary material for: The Impact of Human Mobility on HIV Transmission in Kenya
Source: PLoS One. 2015 Nov 24;10(11):e0142805. doi: 10.1371/journal.pone.0142805 (PMC4657931; doi:10.1371/journal.pone.0142805)
Supplement: S2 Table — (PDF) [file pone.0142805.s002.pdf]

a	b	c	d	e	f	g
Region	Regional population (P_i)	Population aged(15-64) $N_i = 0.548P_i$	Infected (J_i)	Susceptible population $S_i = N_i - J_i$	Removed population ($R_i = 0.13 * J_i$)	Infectious population $I_i = J_i - R_i$
Kirinyaga	1,470,635	805,467	30,608	774,859	3,979	26,629
Embu	1,907,298	1,044,627	28,311	1,016,316	3,680	24,630
Laikipia	995,495	545,233	27,716	517,517	3,603	24,113
Nairobi	5,860,235	3,209,651	216,764	2,992,887	28,179	188,585
Trans Nzoia	2,563,818	1,404,203	83,985	1,320,218	110,918	73,067
Busia	2,192,747	1,200,968	70,554	11,130,413	9,172	61,382
Marsabit	1,057,520	579,204	14,596	564,607	1,898	12,699
Narok	1,406,481	770,330	53,923	716,407	7,010	46,913
West Pokot	1,368,089	749,302	58,067	691,235	7,549	50,518
Taita Taveta	971,969	532,347	38,667	493,680	7,549	33,641
Nakuru	2,959,626	1,620,987	96,384	1,524,604	12,530	83,854
Mombasa	1,589,301	870,460	40,912	829,549	5,319	35,593
Kisumu	3,802,651	2,082,712	217,032	1,865,680	28,214	188,818
Lamu	341,614	187,102	14,781	172,321	1,922	1,2860
Siaya	2,502,955	1,370,868	116,970	1,253,899	15,206	101,764
Wajir	661,941	362,545	3,625	358,920	471	3,154
Kitui	1,897,236	1,039,116	48,838	99,0278	6,349	42,489
Homa Bay	3,033,246	1,661,309	254,180	1,407,129	33,043	221,137
Nyeri	917,505	502,517	35,176	467,341	4,573	30,603
Kilifi	1,109,735	607,802	48,016	559,786	6,242	41,774
Total	38,610,097	21,146,750	1,499,106	19,647,644	194,884	1,304,222

S2 Table:(a) The population of Kenya from the 2009 Census. In the census, there are 47 counts, but in the data of Wesolowski et al [3] there are only 20 regions. We used geographic overlap to combine together groups of regions and come up with 20 regions for this study. The twenty regions are combined as follows: 1. Kirinyaga (3.8) is combined with Murang'a (3.8); 2. Embu (4.7) is combined with Tharaka (4.7) and Mandera (1.0); 3. Laikipia (7.0) is combined with Nyandarua (3.8); 4. Nairobi (9.0) is combined with Machakos (4.7) and Kiambu (3.8); 5. Trans Nzoia (7.0) is combined with Bungoma (5.1) and Marakwet (7.0); 6. Busia is combined (5.1) with Uasin Gishu (7.0) and Vihiga (5.1); 7. Marsabit (4.7) is combined with Garissa (1.0) and Isiolo (4.7); 8. Narok (7.0) is combined with Baringo (7.0); 9. West Pokot (9.0) is combined with Turkana (7.0); 10. Taita Taveta (7.9) is combined with Kajiado (7.0); 11. Nakuru (7.0) is combined with Meru (4.7); 12. Mombasa (4.7) is combined with Kwale (4.7); 13. Kisumu (15.3) is combined with Nandi (7.0), Kericho (7.0), Bomet (7.0) and Nyamira (15.3); 14. Lamu (7.9) combined with Tana River (7.9); 15. Siaya (15.3) combined with Kakamega (5.1), 16. Wajir (1.0); 17. Kitui (4.7) is combined with Makueni (4.7); 18. Homa Bay is combined with Kisii (15.5) and Migori (15.3); 19. Nyeri (3.8) is combined with Samburu (7.8); 20. Kilifi (7.9). The number in the parentheses represents the HIV prevalence of the county [5].
(b) Represents the population sum of the region i, following the combinations of regions in (a) above [1, 2].
(c) Represents the adult populations. The conversions are made by taking the percentage of adult population to be 54.77 for all region [4].
(d) Represents the total number of infected individuals in a region. The infections is contributed by the HIV prevalences of each count constituting the named region.
(e) Represents the susceptible individuals of each region.
(f) Represents the number of the removed class for each region. The removed individuals are those whose CD4 count is less than 200 and are no longer sexually active. The total percentage of HIV infected individuals who are not in treatment with CD4 counts less than 200 in Kenya is 13.3% [5].
(g) Represents the number of infected and infectious individuals in each region.

References

[1] Kenya, S. Country-counties in Kenya; 2013.

[2] Oparanya WA. Population and housing census results. Kenya Census;2009.

[3] Wesolowski A, Eagle N, Tatem AJ , Smith DL, Noor AM, Snow RW, et al. Supplementary Material on the Quantifying the impact of human mobility on Malaria. Science. 2012 338(6104): 267–270.

[4] David Z, Claros E, Africa HDI, Sthrenberg J, Market selection Ind, Julia S, Hiroko M, DataBank Home, and Corporate Procurement. Explore. Create. Share: Development Data.

[5] Kenya, A.I.D.S. Indicator Survey 2007 Nairobi Kenya: National AIDS and STI Control Programme. Ministry of Health Kenya (NASCOP); 2008.
