# Supplementary material for: The Impact of Human Mobility on HIV Transmission in Kenya
Source: PLoS One. 2015 Nov 24;10(11):e0142805. doi: 10.1371/journal.pone.0142805 (PMC4657931; doi:10.1371/journal.pone.0142805)
Supplement: S3 Table — (PDF) [file pone.0142805.s003.pdf]

S3 Table: The monthly average number of trips per individuals between all pairs of 20 regions over the course of the year.

A

	1	2	3	4	5	6	7	8	9	10
1	0	0	0.082	0.98	0.021	0.001	0.001	0.034	0.001	0.004
2	0.02	0	0.003	0.09	0.008	0.008	0.008	0.005	0.001	0.001
3	0.114	0	0	0.168	0.223	0	0	0.153	0.001	0.002
4	0.537	0.002	0.063	0	0.103	0.003	0.006	0.169	0.008	0.022
5	0.022	0	0.113	0.176	0	0	0	0.029	0.012	0.003
6	0.068	0.01	0.01	0.19	0.011	0	0.004	0.013	0.002	0.002
7	0.013	0.002	0.002	0.064	0.005	0	0	0.003	0.004	0.002
8	0.112	0	0.336	1.096	0.071	0.001	0.001	0	0.002	0.007
9	0.016	0.001	0.007	0.085	0.186	0.001	0.004	0.007	0	0.001
10	0.045	0	0.089	0.339	0.041	0.001	0.002	0.019	0.002	0
11	0.459	0.002	0.014	0.256	0.013	0.003	0.003	0.014	0.001	0.003
12	0.08	0.002	0.015	0.434	0.046	0.001	0.006	0.036	0.004	0.088
13	0.012	0	0.088	0.123	0.204	0	0	0.063	0.001	0.005
14	0.034	0	0.008	0.13	0.012	0	0.023	0.01	0.001	0.021
15	0.014	0	0.011	0.159	0.215	0	0	0.014	0.001	0.002
16	0.017	0.008	0.002	0.068	0.006	0.005	0.013	0.003	0	0.001
17	0.134	0	0.004	0.625	0.007	0	0.001	0.008	0.03	0.091
18	0.011	0	0.006	0.158	0.028	0	0	0.031	0	0.002
19	0.279	0	0.134	0.102	0.028	0.02	0	0.02	0	0.003
20	0.02	0	0.004	0.089	0.01	0	0.002	0.007	0.001	0.033

B

	11	12	13	14	15	16	17	18	19	20
1	0.207	0.033	0.015	0.002	0.024	0.001	0.051	0.014	0.059	0.002
2	0.019	0.014	0.005	0.002	0.009	0.028	0.004	0.005	0.001	0.001
3	0.011	0.009	0.093	0.001	0.028	0	0.004	0.013	0.025	0.001
4	0.075	0.116	0.101	0.007	0.276	0.002	0.222	0.149	0.006	0.007
5	0.007	0.022	0.163	0.001	0.615	0	0.006	0.044	0.003	0.001
6	0.098	0.019	0.01	0.002	0.016	0.023	0.005	0.01	0.038	0.001
7	0.01	0.023	0.003	0.026	0.006	0.004	0.003	0.004	0	0.003
8	0.024	0.028	0.168	0.003	0.091	0	0.02	0.092	0.005	0.002
9	0.007	0.011	0.014	0.001	0.037	0	0.004	0.009	0.001	0.001
10	0.015	0.393	0.138	0.014	0.033	0.001	0.272	0.019	0.011	0.051
11	0	0.03	0.009	0.002	0.014	0.002	0.015	0.009	0.011	0.002
12	0.034	0	0.048	0.07	0.145	0.002	0.148	0.059	0.002	0.2

										34
13	0.004	0.014	0	0.001	0.369	0	0.004	0.388	0.001	0.001
14	0.017	0.45	0.01	0	0.022	0	0.02	0.013	0.001	0.229
15	0.004	0.029	0.202	0.001	0	0	0.005	0.076	0	0.001
16	0.028	0.012	0.004	0.001	0.006	0	0.003	0.003	0	0.001
17	0.019	0.077	0.007	0.002	0.01	0	0	0.006	0.001	0.004
18	0.003	0.02	0.35	0.001	0.134	0	0.004	0	0.001	0.001
19	0.07	0.009	0.01	0.001	0.013	0	0.003	0.007	0	0.001
20	0.012	0.768	0.009	0.338	0.018	0	0.019	0.013	0.001	0

Source: Wesolowski et al (2012) S2 Table: We have divide by 1,000 the original data to get the average trips per year per each individual.

References:

Wesolowski A, Eagle N, Tatem AJ , Smith DL, Noor AM, Snow RW, Buckee CO (2012) Supplementary Material on the Quantifying the impact of human mobility on Malaria. Science 338(6104): 267--270.
