# Supplementary material for: The Impact of Human Mobility on HIV Transmission in Kenya
Source: PLoS One. 2015 Nov 24;10(11):e0142805. doi: 10.1371/journal.pone.0142805 (PMC4657931; doi:10.1371/journal.pone.0142805)
Supplement: S4 Table — (PDF) [file pone.0142805.s004.pdf]

The 47 Counties Population and HIV prevalence in Kenya.

Code	County	Former Province	Population Census 2009	Capital	HIV Prevalence(%)
	Totals		38,610,097		
	Sub-Totals	Central	4383743		
	Sub-Totals	Coast	3325307		
	Sub-Totals	Eastern	5668123		
	Sub-Totals	North Eastern	2310757		
	Sub-Totals	Nyanza	5442711		
	Sub-Totals	Rift Valley	10006805		
	Sub-Totals	Western	4334202		
1	Mombasa	Coast	939370	Mombasa (City)	7.9
2	Kwale	Coast	649931	Kwale	7.9
3	Kilifi	Coast	1109735	Kilifi	7.9
4	Tana River	Coast	240075	Hola	7.9
5	Lamu	Coast	101539	Lamu	7.9
6	Taita-Taveta	Coast	284657	Voi	7.9
7	Garissa	North Eastern	623060	Garissa	1
8	Wajir	North Eastern	661941	Wajir	1
9	Mandera	North Eastern	1025756	Mandera	1
10	Marsabit	Eastern	291166	Marsabit	4.7
11	Isiolo	Eastern	143294	Isiolo	4.7
12	Meru	Eastern	1356301	Meru	4.7
13	Tharaka-Nithi	Eastern	365330	Chuka	4.7
14	Embu	Eastern	516212	Embu	4.7
15	Kitui	Eastern	1012709	Kitui	4.7
16	Machakos	Eastern	1098584	Machakos	4.7
17	Makueni	Eastern	884527	Wote	4.7
18	Nyandarua	Central	596268	Ol Kalou	3.8
19	Nyeri	Central	693558	Nyeri	3.8
20	Kirinyaga	Central	528054	Kerugoya / Kutus	3.8
21	Murang'a	Central	942581	Murang'a	3.8
22	Kiambu	Central	1623282	Kiambu	3.8
23	Turkana	Rift Valley	855399	Lodwar	7
24	West Pokot	Rift Valley	512690	Kapenguria	7
25	Samburu	Rift Valley	223947	Maralal	7
26	Trans Nzoia	Rift Valley	818757	Kitale	7
27	Uasin Gishu	Rift Valley	894179	Eldoret	7

28	Elgeyo-Marakwet	Rift Valley	369998	Iten	7
29	Nandi	Rift Valley	752965	Kapsabet	7
30	Baringo	Rift Valley	555561	Kabarnet	7
31	Laikipia	Rift Valley	399227	Rumuruti	7
32	Nakuru	Rift Valley	1603325	Nakuru	7
33	Narok	Rift Valley	850920	Narok	7
34	Kajiado	Rift Valley	687312	Kajiado	7
35	Kericho	Rift Valley	752396	Kericho	7
36	Bomet	Rift Valley	730129	Bomet	7
37	Kakamega	Western	1660651	Kakamega	5.1
38	Vihiga	Western	554622	Vihiga	5.1
39	Bungoma	Western	1375063	Bungoma	5.1
40	Busia	Western	743946	Busia	5.1
41	Siaya	Nyanza	842304	Siaya	15.3
42	Kisumu	Nyanza	968909	Kisumu	15.3
43	Homa Bay	Nyanza	963794	Homa Bay	15.3
44	Migori	Nyanza	917170	Migori	15.3
45	Kisii	Nyanza	1152282	Kisii	15.3
46	Nyamira	Nyanza	598252	Nyamira	15.3
47	Nairobi	Nairobi	3138369	Nairobi (City)	9
Total			38610097		

Source: https://en.wikipedia.org/wiki/Counties_of_Kenya and The National AIDS and STI Control Programme (2008) Kenya AIDS indicator survey 2007.
