# Supplementary material for: The Impact of Human Mobility on HIV Transmission in Kenya
Source: PLoS One. 2015 Nov 24;10(11):e0142805. doi: 10.1371/journal.pone.0142805 (PMC4657931; doi:10.1371/journal.pone.0142805)
Supplement: S6 Table — (PDF) [file pone.0142805.s006.pdf]

S6 Table: The average number of trips per month between all pairs of 20 regions. For clarity, we have divided the movement into two tables displaying the average number of monthly trips (A) from regions 1-20 to regions 1-10, (B) from regions 1-20 to regions 11-20 over the course of the year.

A

	1	2	3	4	5	6	7	8	9	10
1	0	1,503	266,134	3,173,897	66,796	3,733	4,087	108,656	3,044	13,817
2	7,188	0	1,049	32,574	3,002	2,790	2,877	1,866	354	387
3	239,738	178	0	352,498	469,162	605	363	321,193	1,338	5,117
4	3,233,013	14,695	377,539	0	623,208	18,189	36,801	1,017,159	47,502	135,104
5	59,130	671	298,360	464,425	0	815	985	76,640	31,411	6,607
6	17,824	2,601	2,651	49,601	2,926	0	998	3,474	581	472
7	8,393	1,359	1,158	41,705	3,005	213	0	2,036	2,576	1,248
8	126,317	328	379,127	1,235,222	80,506	1,064	1,135	0	1,766	7,499
9	7,249	277	3,269	39,167	85,947	589	1,739	3,082	0	531
10	16,519	160	32,407	123,338	14,950	208	646	6,973	604	0
11	771,849	2,528	23,372	429,592	21,150	5,109	4,783	23,582	1,049	4,610
12	144,927	2,934	27,703	786,120	83,375	1,442	10,525	65,209	7,289	159,922
13	35,280	396	261,839	366,631	607,408	675	535	188,254	1,714	13,465
14	17,055	103	3,782	65,438	5,804	148	11,448	4,932	440	10,307
15	82,775	800	62,491	944,903	1,277,344	1,124	1,397	82,949	4,937	12,153
16	7,847	3,524	906	31,481	2,852	2,145	5,841	1,383	136	557
17	202,134	226	6,199	944,706	10,904	429	1,131	12,828	44,680	138,038
18	36,061	538	20,120	520,634	91,975	611	960	101,923	1,196	5,560
19	94,667	49	45,446	34,497	9,347	6,666	76	6,647	126	914
20	10,447	84	2,111	46,355	5,175	104	930	3,463	275	17,127

B

	11	12	13	14	15	16	17	18	19	20
1	670,037	108,288	48,250	7,203	76,910	1,693	164,472	44,827	191,959	6,589
2	6,810	5,132	1,672	660	3,215	10,158	1,382	1,752	315	378
3	22,377	18,757	196,515	1,449	59,862	246	7,445	26,686	51,837	1,342
4	452,223	698,256	610,101	40,986	1,660,809	15,013	1,340,047	895,662	33,856	43,945
5	17,924	56,916	430,259	2,815	1,620,008	714	14,741	116,354	8,370	3,124
6	25,720	4,996	2,641	458	4,203	5,893	1,181	2,629	9,854	337
7	6,410	14,743	1,997	17,133	4,192	2,806	1,929	2,827	157	1,862
8	27,430	31,201	189,830	3,047	102,367	445	22,160	103,603	5,129	2,331
9	3,203	4,881	6,267	372	17,215	214	1,927	3,975	529	331
10	5,313	143,142	50,256	5,100	11,884	215	98,988	6,999	3,977	18,480
11	0	51,075	15,089	3,701	23,832	3,880	25,368	14,689	18,979	3,061
12	61,370	0	86,216	126,710	262,464	3,813	268,840	106,727	4,224	423,514
13	12,223	41,938	0	1,989	1,101,371	395	10,712	1,156,828	1,651	2,463
14	8,672	226,063	5,205	0	11,073	178	10,160	6,551	490	115,341
15	24,381	172,658	1,201,947	4,727	0	847	29,353	450,648	2,301	8,066
16	12,848	5,559	1,846	659	2,908	0	1,188	1,478	177	422
17	28,964	115,701	9,900	3,158	15,139	296	0	8,421	917	5,418
18	11,420	65,014	1,153,673	2,301	440,772	440	11,594	0	1,651	3,085
19	23,867	2,933	3,462	260	4,275	87	922	2,473	0	196
20	6,228	398,564	4,675	175,365	9,499	144	10,106	6,834	527	0

Source: Wesolowski et al (2012) S4 Table.
