# Supplementary material for: The Impact of Human Mobility on HIV Transmission in Kenya
Source: PLoS One. 2015 Nov 24;10(11):e0142805. doi: 10.1371/journal.pone.0142805 (PMC4657931; doi:10.1371/journal.pone.0142805)
Supplement: S1 Figs — (PDF) [file pone.0142805.s007.pdf]

S1 Figs: Relevant Figures incorporated in the article "The Impact of Human Mobility on HIV Transmission in Kenya"

Augustino Isdory[1,*], Eunice W. Mureithi [1,¶], David J. T. Sumpter[2,¶]

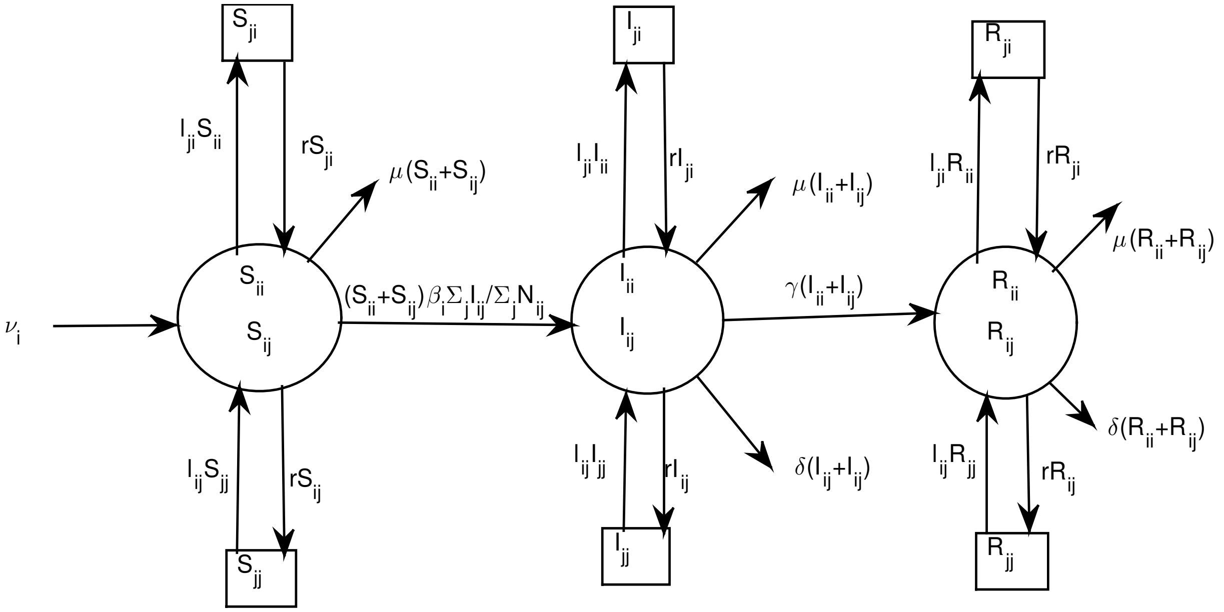

S1 Fig 1. Flow diagram between regions for the SIR model with n-patches. For clarity, we consider individuals travelling to another region after returning home first.

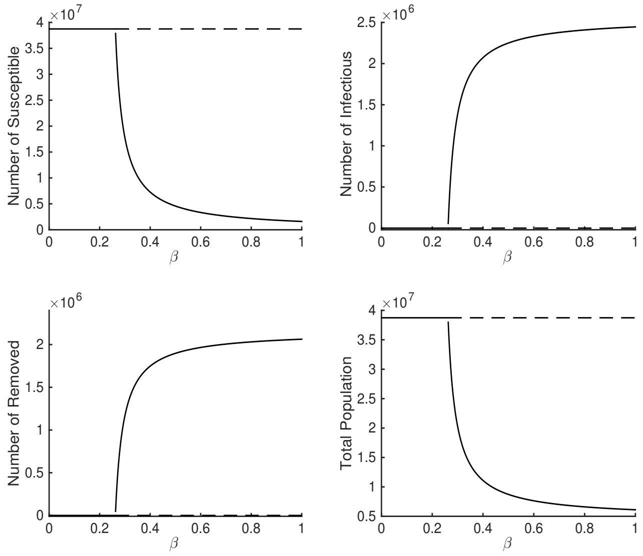

S1 Fig 2. Bifurcation diagram of the of the system without regions and mobility. The solid and dotted lines show the values at which the disease-free equilibrium point is stable and unstable respectively. The solid curves show the values at which the endemic equilibrium point is stable.

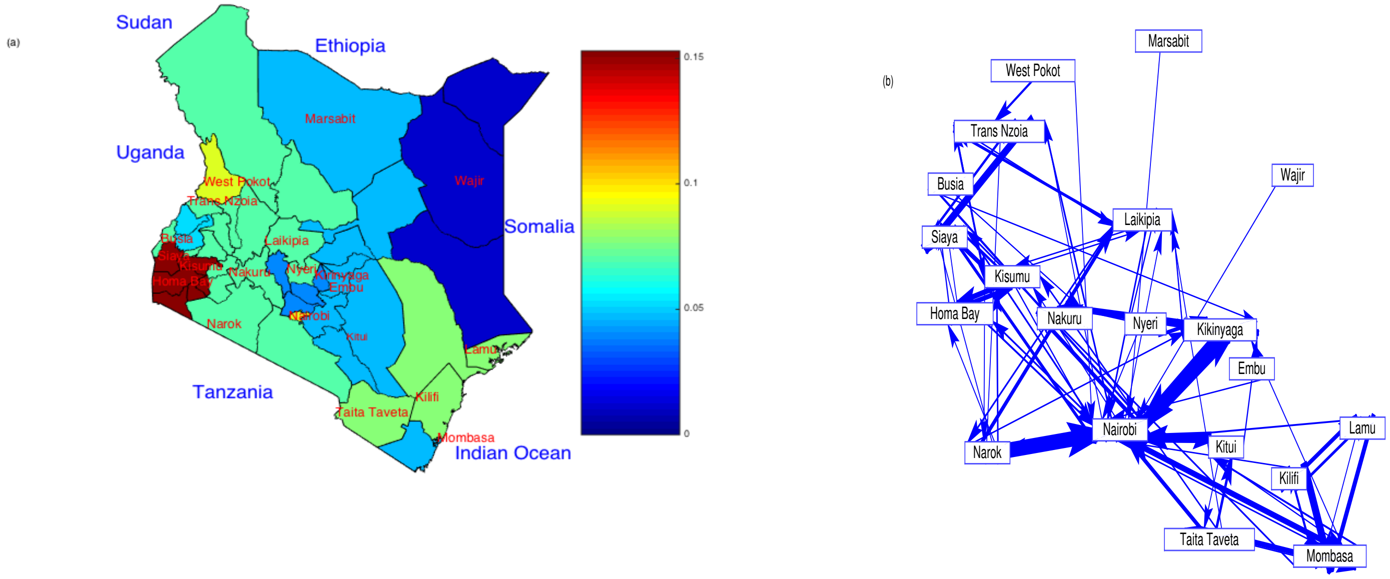

S1 Fig 3. (a) Map of Kenya showing HIV prevalence distributions. The color bar from blue to red is in the order of increasing HIV prevalence. For clarity, the names of counties included are the only ones included in this study (Source of data:ArcGIS.com: shapefile-The 47 counties of Kenya (shapefile by dmuthami S5 Table) and HIV data from KAIS. (b) Human travel networks (S6 Table) as estimated by Wesolowski et al. Monthly average number of trips per 1000 individuals between all pairs of regions over the course of the year. For clarity, only trips made per 1000 individuals that are more than 60 trips per year are shown, with arrows indicating the direction of movements from home region to a visited region. The thickness of the arrow represents the number of trips made.

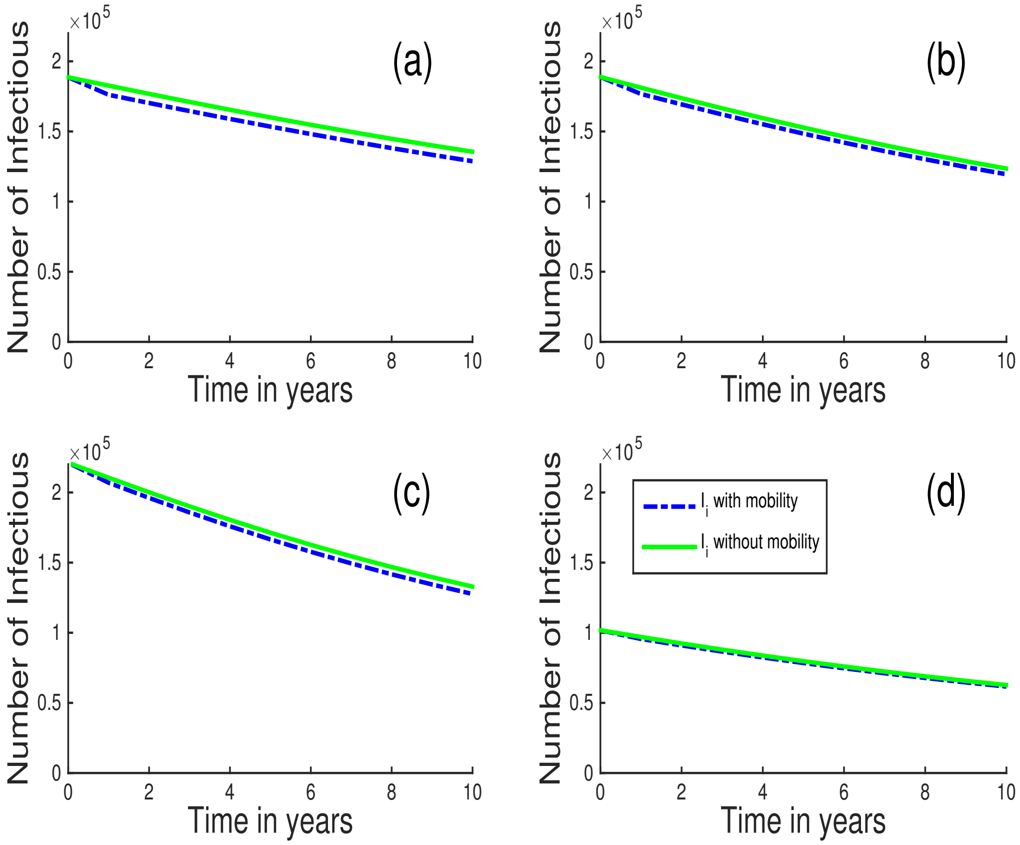

S1 Fig 4. Time evolution of the metapopulation model for (a) Nairobi, (b) Kisumu, (c) Homa Bay and (d) Siaya for $R_B < 1$. The dotted blue lines represent infectious individuals when there is human mobility and the solid green lines represent infectious individuals when there is no human mobility between the regions.

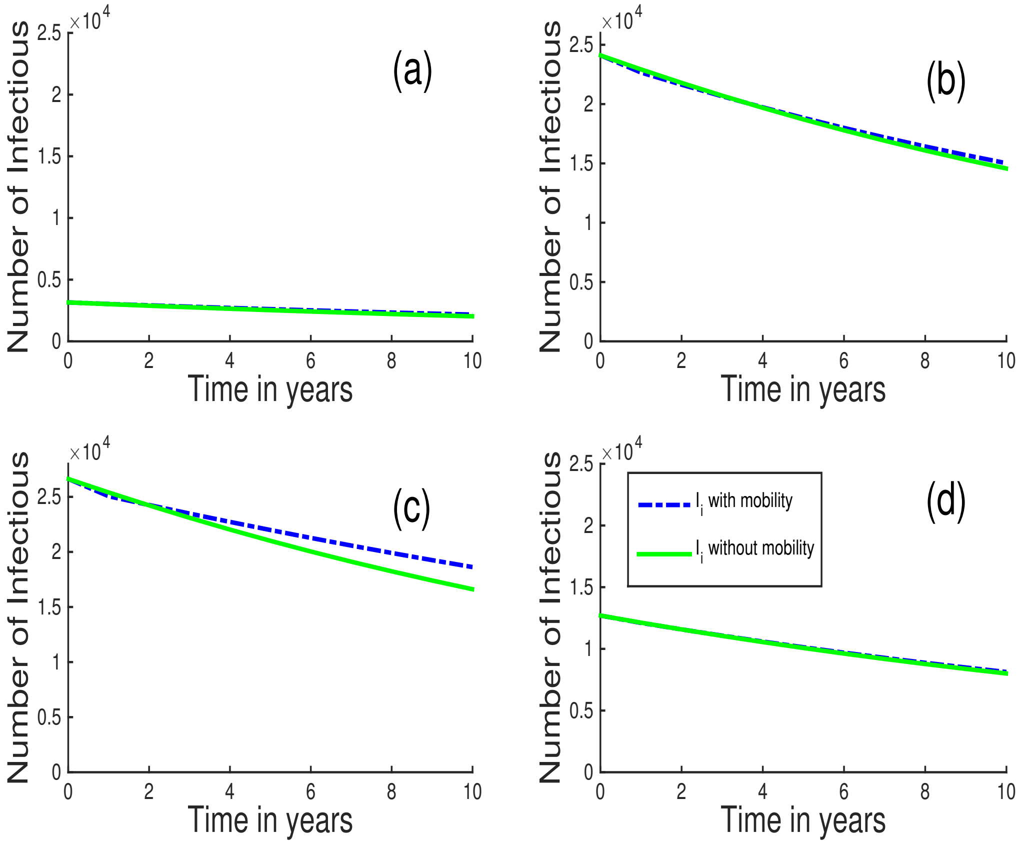

S1 Fig 5. Time evolution of the metapopulation model for (a) Wajir, (b) Laikipia, (c) Kirinyaga, (d) Marsabit for $R_B < 1$. The dotted blue lines represent infectious individuals when there is human mobility and the solid green lines represent infectious individuals when there is no human mobility between the regions.

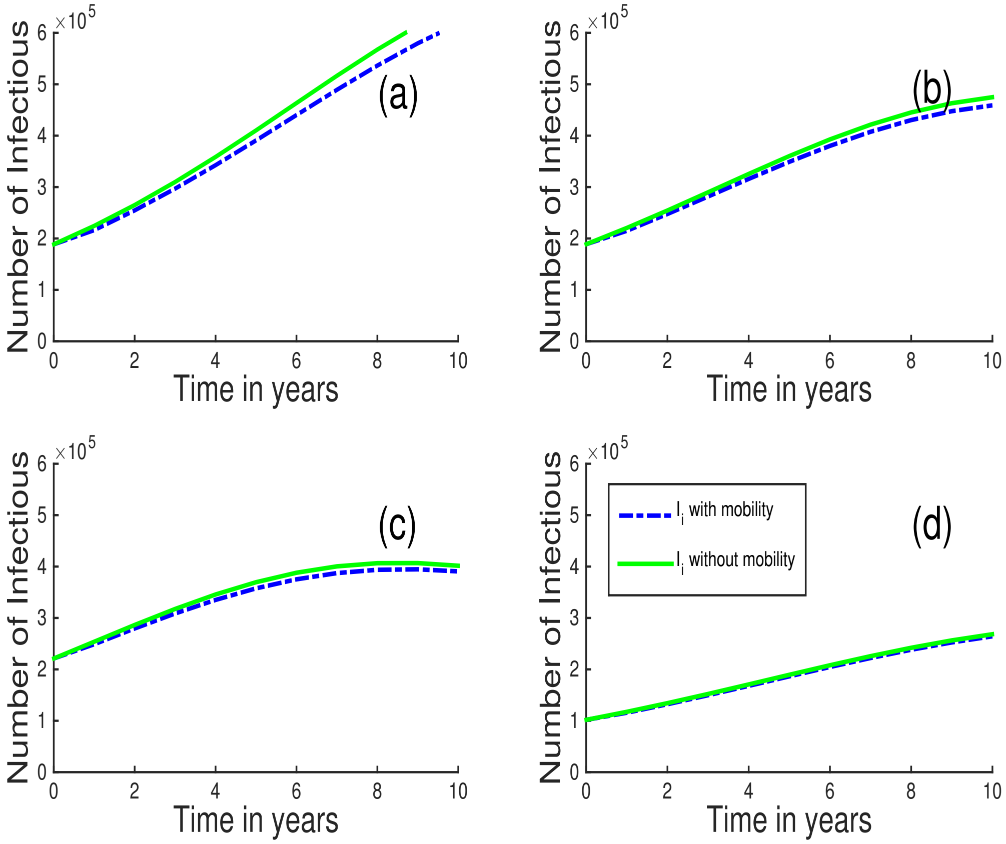

S1 Fig 6. Time evolution of the metapopulation model for (a) Nairobi, (b) Kisumu, (c) Homa Bay and (d) Siaya for $R_B > 1$. The dotted blue represent infectious individuals when there is human mobility and the solid green lines represent Infectious individuals when there is no human mobility between the regions.

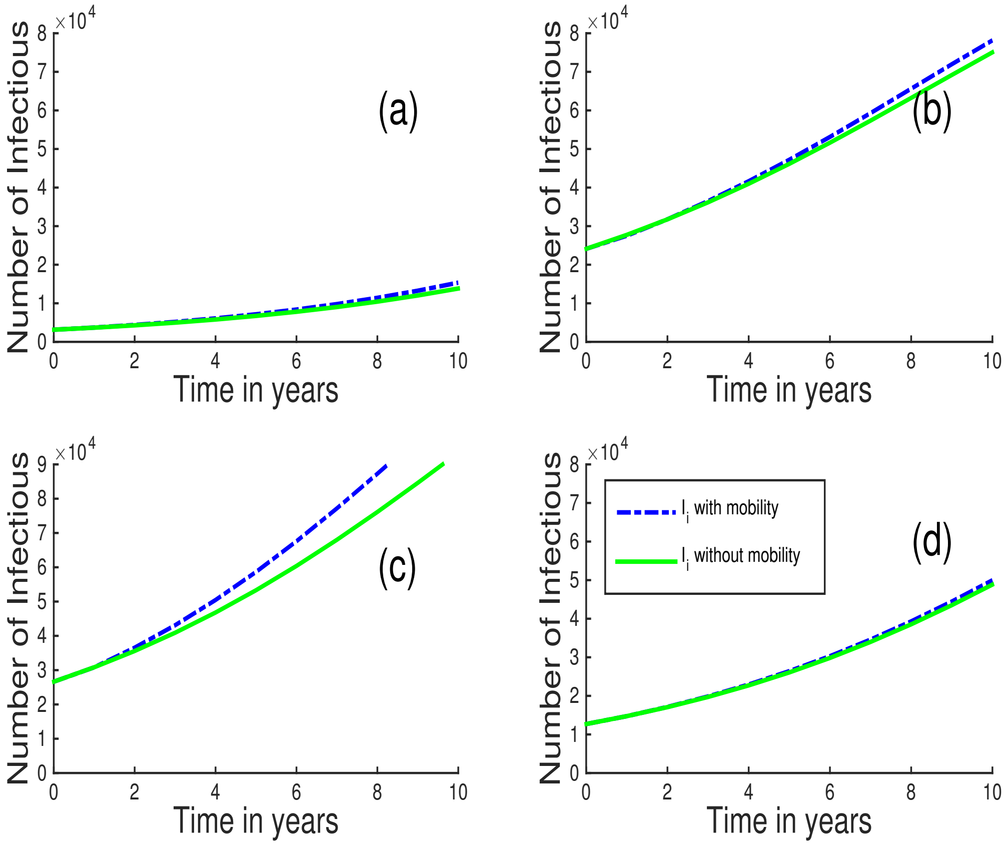

S1 Fig 7. Time evolution of the metapopulation model for (a) Wajir, (b) Laikipia, (c) Kirinyaga, (d) Marsabit for $R_B > 1$. The dotted blue lines represent infectious individuals when there is human mobility and the solid green lines represent Infectious individuals when there is no human mobility between the regions.

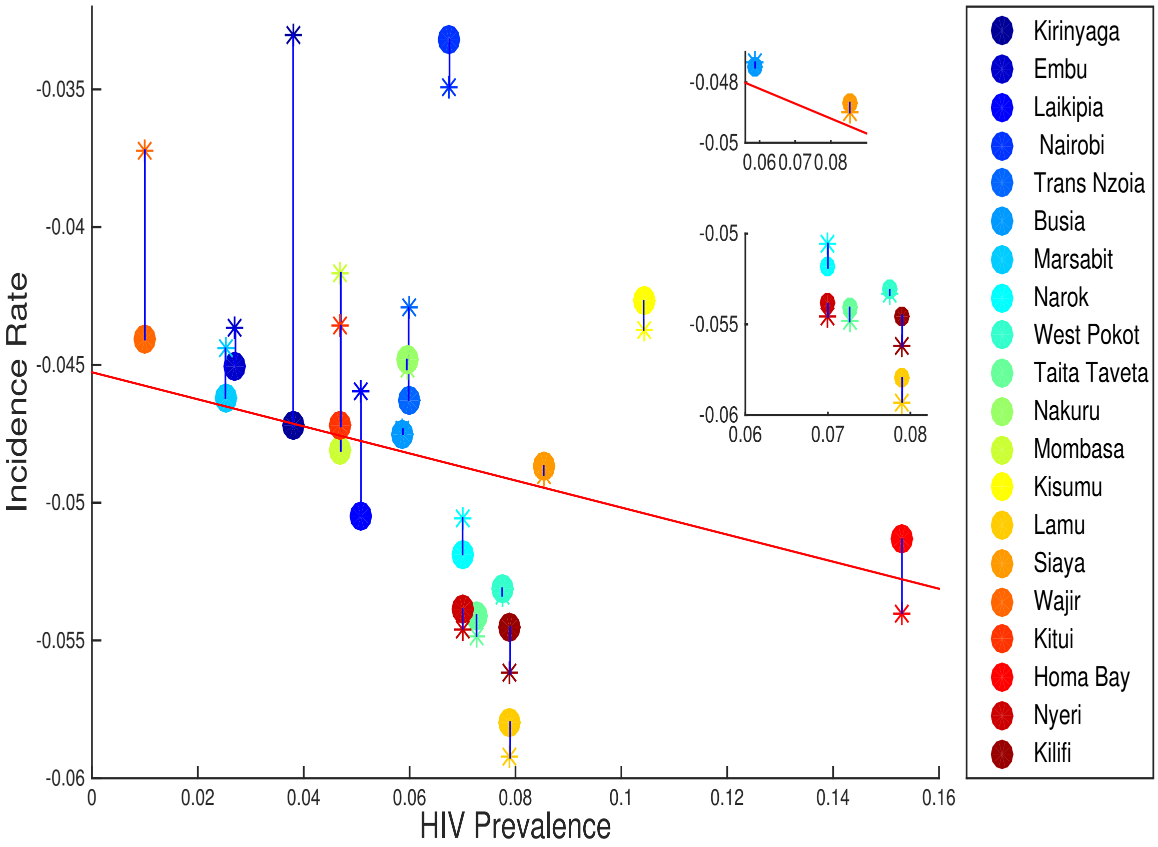

S1 Fig 8. Incidence rate versus prevalence rate. A dot represents the dynamics of HIV infections without human mobility between the regions and a star represents the dynamics of HIV infections when there is human mobility between the regions for $R_B < 1$.

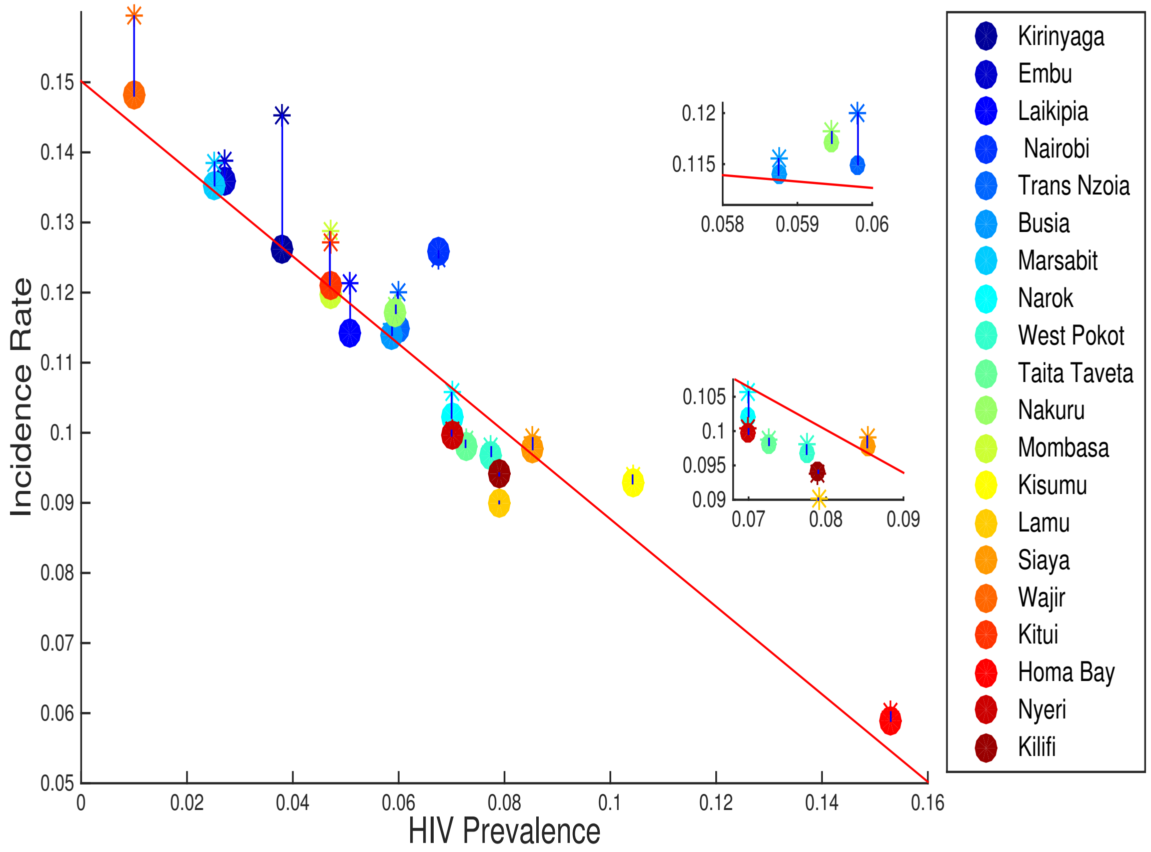

S1 Fig 9. Incidence rate versus prevalence rate. A dot represents the dynamics of HIV infections without human mobility between the regions and a star represents the dynamics of HIV infections when there is human mobility between the regions for $R_B > 1$.

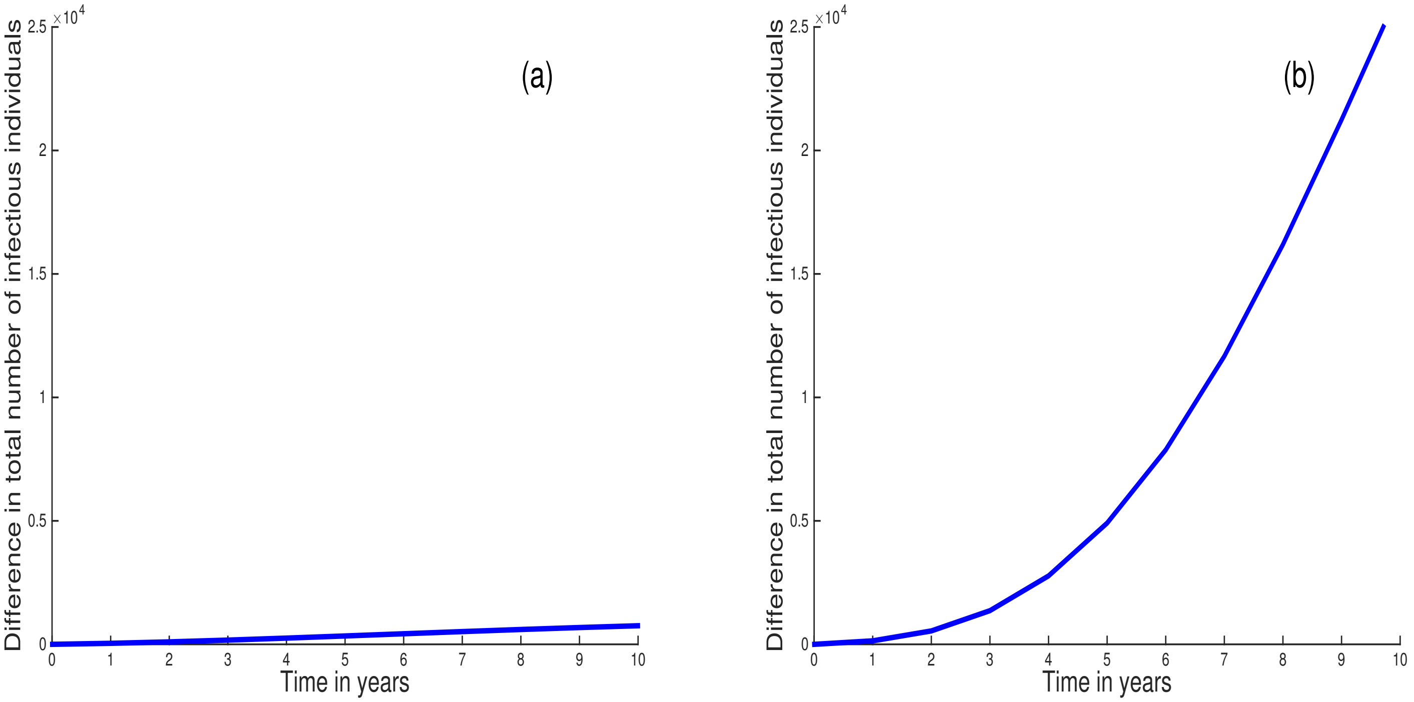

S1 Fig 10. (a) and (b) show the differences between the total number of infectious individuals when there is human mobility and when there is no human mobility within the regions in the country for $R_B < 1$ and $R_B > 1$ respectively.
